# Clonal hematopoiesis dynamics influences long‐term outcomes of follicular lymphoma: Results from FIL FOLL12 trial

**DOI:** 10.1002/hem3.70393

**Published:** 2026-05-20

**Authors:** Nawar Maher, Riccardo Moia, Mohammad Almasri, Luca Cividini, Elisa Genuardi, Chiara Cosentino, Roberta Soscia, Giovanni Manfredi Assanto, Rita Tavarozzi, Maria Carmela Vegliante, Luisa Lorenzi, Annalisa Andorno, Luca Arcaini, Simone Ragaini, Benedetta Puccini, Caterina Patti, Armando Santoro, Gloria Margiotta‐Casaluci, Vittorio Ruggero Zilioli, Manuela Zanni, Sonia Ronconi, Francesco Di Raimondo, Annalisa Arcari, Catello Califano, Claudia Castellino, Annarita Conconi, Tommasina Perrone, Donato Mannina, Caterina Plenteda, Francesco Alesiani, Francesca Gaia Rossi, Angelo Michele Carella, Luigi Marcheselli, Sara Galimberti, Sabino Ciavarella, Riccardo Bomben, Ilaria Del Giudice, Marco Ladetto, Gianluca Gaidano, Stefano Luminari, Simone Ferrero

**Affiliations:** ^1^ Division of Hematology, Department of Translational Medicine Università del Piemonte Orientale and AOU Maggiore della Carità di Novara Novara Italy; ^2^ Department of Translational Medicine, SCDU Ematologia AO SS Antonio e Biagio E Cesare Arrigo Università del Piemonte Orientale Alessandria Italy; ^3^ Division of Hematology, Department of Molecular Biotechnologies and Health Sciences University of Torino Torino Italy; ^4^ Hematology, Department of Translational and Precision Medicine Sapienza University of Rome – AOU Policlinico Umberto I Roma Italy; ^5^ Hematology and Cell Therapy Unit IRCCS Istituto Tumori 'Giovanni Paolo II Bari Italy; ^6^ Pathology Unit, Department of Molecular and Translational Medicine University of Brescia Brescia Italy; ^7^ Division of Pathology, Department of Health Sciences Università del Piemonte Orientale and Ospedale Maggiore della Carità Novara Italy; ^8^ Department of Molecular Medicine University of Pavia & Division of Hematology, Fondazione IRCCS Policlinico San Matteo Pavia Italy; ^9^ Hematology Unit Careggi University Hospital Firenze Italy; ^10^ Ospedali Riuniti Villa Sofia ‐ Cervello (Presidio V. Cervello) Palermo Italy; ^11^ Department of Oncology and Haematology IRCCS Humanitas Research Hospital Rozzano Italy; ^12^ Division of Hematology ASST Grande Ospedale Metropolitano Niguarda Milano Italy; ^13^ IRCCS Istituto Romagnolo per lo studio dei Tumori “Dino Amadori” – IRST S.R.L, Ematologia Meldola Italy; ^14^ Azienda Ospedaliera Universitaria Policlinico ‐ S. Marco, UOC di Ematologia & University of Catania Catania Italy; ^15^ Azienda USL Piacenza, UOC Ematologia e Centro Trapianti Piacenza Italy; ^16^ Presidio ospedaliero “A. TORTORA”, U.O. Onco‐ematologia Pagani Italy; ^17^ A.O. S. Croce e Carle, S.C. Ematologia Cuneo Italy; ^18^ Azienda Sanitaria Locale, Ematologia, Ospedale degli Infermi Biella Italy; ^19^ AOU Policlinico Consorziale, U.O. Ematologia con Trapianto Bari Italy; ^20^ Azienda Ospedali Riuniti Papardo‐Piemonte, S.C. Ematologia Messina Italy; ^21^ AOU di Parma, UOC Ematologia e CTMO Parma Italy; ^22^ ASUR 8, Medicina Interna e Ematologia, Civitanova Marche Italy; ^23^ Ospedale Maggiore Policlinico ‐ Fondazione IRCCS Ca Granda, Ematologia Milano Italy; ^24^ Ematologia e Unità Terapia Intensiva Ematologica, IRCCS Fondazione Casa Sollievo della Sofferenza San Giovanni Rotondo (FG) San Giovanni Rotondo Italy; ^25^ Uffici Studi Fondazione Italiana Linfomi Modena Italy; ^26^ Hematology Division Pisa University Hospital, Department of Clinical and Experimental Medicine, University of Pisa Pisa Italy; ^27^ Centro di Riferimento Oncologico di Aviano Aviano Italy; ^28^ Ematologia, Azienda Unità Sanitaria Locale – IRCCS Reggio Emilia Reggio Emilia Italy; ^29^ CHIMOMO Department University of Modena and Reggio Emilia Modena Italy

## Abstract

Whether clonal hematopoiesis (CH) in follicular lymphoma (FL) patients affects clinical outcome or is merely a bystander phenomenon is unclear. We leveraged the Phase III Fondazione Italiana Linfomi FOLL12 trial, which treated patients with advanced‐stage FL with R‐CHOP or R‐Bendamustine, to evaluate the role of myeloid CH at baseline and after chemoimmunotherapy (CIT). A total of 528 serial blood samples from 242 FL were analyzed by CAPP‐Seq. At baseline, CH occurred in 35.5% patients with *DNMT3A* (*N* = 41, 16.9%) and *TET2* (*N* = 29, 12.0%) being the most frequently mutated genes. After a median follow‐up of 8.2 years, CH at baseline did not impact progression‐free survival (PFS), overall survival (OS), or risk of transformation (P = 0.660, P = 0.230, and P = 0.584, respectively), but instead associated with therapy‐related hematological toxicities driven by *TET2* mutations. CH dynamics after the genotoxic pressure imposed by CIT was evaluated in 211 patients provided with sequential samples. CIT significantly expanded both prevalence and size of CH, with clones affected by DNA damage response (DDR) gene mutations exhibiting the highest fitness. Distinct selective pressures were observed between R‐CHOP and R‐Bendamustine, with the latter creating a tighter bottleneck that facilitates the emergence of fitter CH clones preferentially carrying *TP53* mutations. Patients acquiring fit DDR clones (*N* = 37) had inferior long‐term outcomes, including independent increased risk of second malignancies (hazard ratio [HR] 2.63, P = 0.035) that developed in 28 patients, and shorter OS (HR 3.28, P = 0.008). CH emerges as a novel and potentially valuable biomarker in FL, capable of predicting long‐term toxicities that are key endpoints in indolent lymphoid malignancies characterized by long‐lasting survival.

## INTRODUCTION

Follicular lymphoma (FL) is the most common subtype among indolent non‐Hodgkin lymphomas, and chemoimmunotherapy (CIT), namely CHOP or Bendamustine (Benda) with anti‐CD20 antibodies, still represents today the first‐line standard of care treatment for advanced‐stage disease.^1^, ^2^, ^3^ Since first‐line therapy in FL induces long‐lasting remission in most cases, the identification of biomarkers that can anticipate long‐term toxicities is relevant to maximize survival outcomes and quality of life in FL patients.^4^

Myeloid clonal hematopoiesis (CH) represents an age‐associated expansion of hematopoietic stem or progenitor cells carrying myeloid somatic mutations and frequently involves canonical DTA genes, namely *DNMT3A*, *TET2*, and *ASXL1*.^5^, ^6^, ^7^ However, CH does not evolve uniformly across clinical contexts. In particular, chemotherapy imposes substantial DNA damage on both malignant and non‐malignant hematopoietic stem cells, creating evolutionary bottlenecks that permit the outgrowth of resistant or fitness‐enhanced CH clones, resulting in clonal signatures that are in part distinct from physiological aging.^8^, ^9^, ^10^, ^11^, ^12^, ^13^, ^14^, ^15^, ^16^, ^17^ Within this environment, CH mutations in DNA damage response (DDR) genes, frequently involving *PPM1D*, *TP53*, *CHEK2*, and *RAD21*, often gain a strong advantage, leading to clonal expansion preferentially after treatment.^8^, ^18^ In FL, the two most frequent frontline regimens R‐CHOP and R‐Benda have distinct genotoxic and immunologic profiles, yet how these treatments shape CH clonal trajectories, influence mutation‐specific expansions, or contribute to durable shift in CH architecture is still largely unknown.^19^

Emerging evidence suggests that CH might not be merely a bystander phenomenon in patients with cancer.^20^ Rather, CH may associate with inferior survival and increased risk of therapy‐related neoplasms and may modulate treatment tolerance by altering myeloid function, predisposing to cytopenia and impairing recovery after CIT exposure.^21^, ^22^, ^23^, ^24^, ^25^, ^26^ In this context, we hypothesized that CH at baseline before CIT and its evolutionary dynamics after treatment might represent a determinant of long‐term disease outcomes in FL survivors.

We therefore leveraged the Phase III Fondazione Italiana Linfomi (FIL) FOLL12 trial, which treated patients with advanced‐stage FL with either R‐CHOP or R‐Benda, to evaluate the potential clinical role of myeloid CH at baseline and its evolution after CIT.^27^ Across this cohort, the analysis of 528 serial blood samples from 242 patients provided an unprecedented opportunity to investigate CH and its dynamics and clinical correlations in a Phase III clinical trial for FL. To our knowledge, this represents the first prospective, longitudinal evaluation of CH in newly diagnosed indolent lymphomas.

## MATERIALS AND METHODS

### Patient characteristics

A cohort of 242 patients enrolled in the FIL FOLL12 Phase III clinical trial was assessed to dissect the potential clinical role of CH in FL. The study design and the results of the FOLL12 trial were presented elsewhere.^27^ Briefly, FOLL12 enrolled FL patients requiring first‐line systemic therapy with an induction phase represented by R‐CHOP or R‐Benda, according to the physician's choice. The co‐presence of any overt second primary malignancy, including myeloid neoplasms identified on mandatory bone marrow biopsy performed at enrollment, represented an exclusion criterion from the trial. At enrollment, patients were randomized to receive, after completing induction therapy, standard rituximab maintenance (standard arm) or different maintenance strategies (experimental arm) according to minimal residual disease (MRD) and positron emission tomography (PET)/computed tomography (CT) response. The present ancillary biological study is part of the FIL‐FOLL BIO trial (approved by the Ethical Committee of Torino, prot n. 0066394/2023) and was conducted in accordance with the Declaration of Helsinki. All patients provided written informed consent.

### CH analysis on peripheral blood genomic DNA

Genomic DNA (gDNA) from whole peripheral blood samples was analyzed by next‐generation sequencing‐based Cancer Personalized Profiling by deep sequencing (CAPP‐seq) using a panel of 28 genes frequently mutated in CH (Supporting Information S7: Table 1). In all cases (*N* = 242), gDNA was collected at baseline during the trial screening period; in 211 cases, gDNA was also collected at one or more timepoints during follow‐up to evaluate CH dynamics after induction of CIT. A stringent bioinformatic pipeline for variant calling was used as previously reported.^28^, ^29^ With a median coverage of 3322×, the variant allele frequency (VAF) threshold was set at 1% for variant calling, and CH mutations were confirmed using the CH database that has been recently made available.^30^, ^31^ See also Supporting Information S6: Appendix.

### Clonal fitness and treatment bottleneck effect

Clonal fitness was inferred from paired VAF measurements in baseline and sequential samples using a logistic growth model.^8^, ^17^ To estimate the effective allelic population size ($N_{\text{eff}}$) during treatment, we modeled each CH variant as an independent clone and treated changes in VAF across timepoints as binomial sampling noise.^32^, ^33^ See also Supporting Information S6: Appendix.

### Statistical analysis

Survival analysis for progression‐free survival (PFS) and overall survival (OS) was performed by the Kaplan–Meier method and compared between strata using log‐rank. The Simon–Makuch method was applied to model fit CH clones as a time‐dependent variable. Associations with the development of second malignancies were estimated using both Cox proportional hazards regression and cumulative incidence function (CIF) analysis by Fine‐Gray competing risk modeling, accounting for death as a competing event. Statistics were performed with R version 4.4.2.

## RESULTS

### Patient characteristics

**Figure 1:**
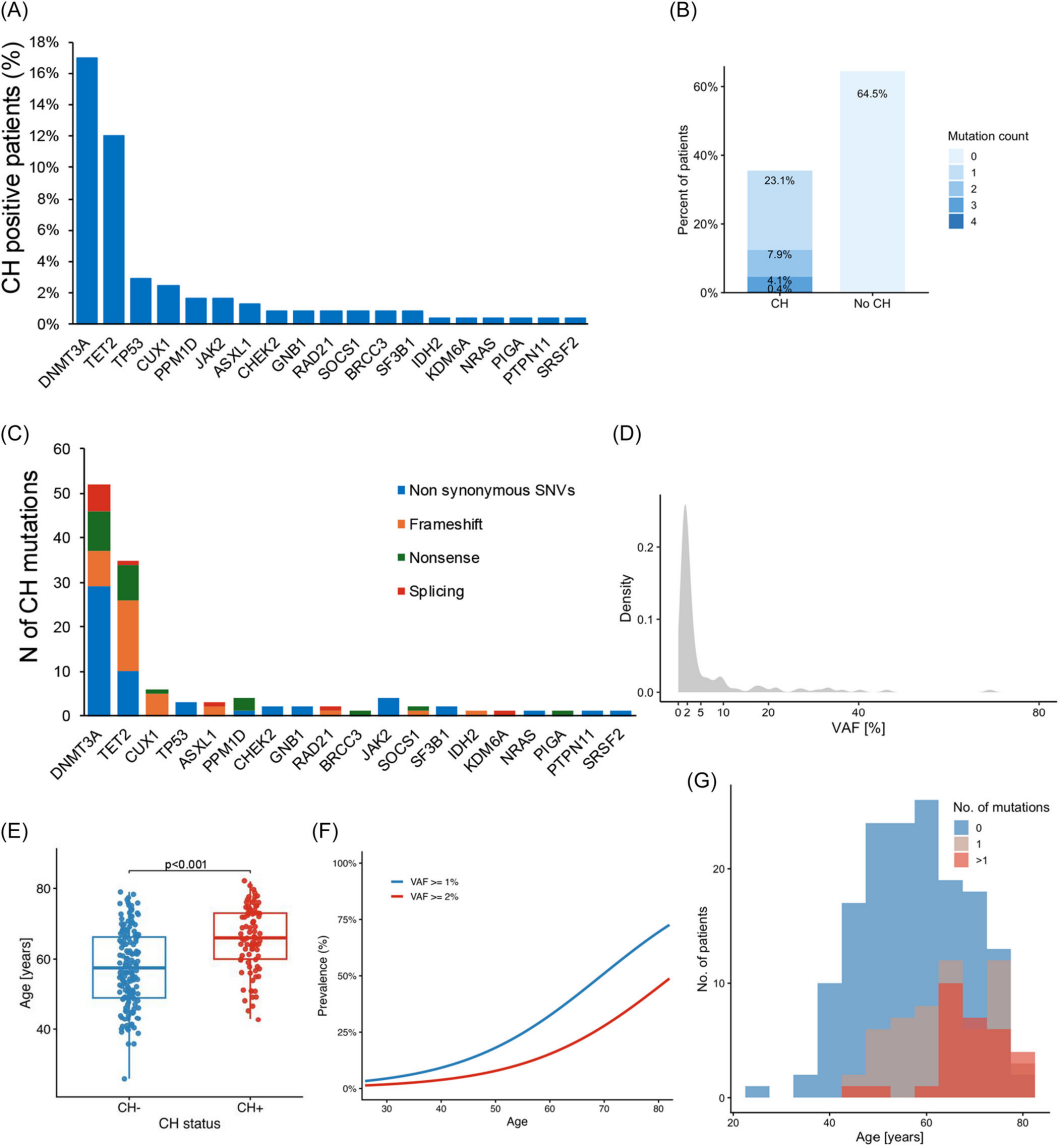
**Clonal hematopoiesis (CH) prevalence and correlation with follicular lymphoma (FL) baseline characteristics. (A)** Bar charts representing the prevalence of the most frequent CH mutations. **(B)** Percentage of patients with and without CH stratified by the number of mutations (0–4). **(C)** Bar charts representing the different types of CH mutations. **(D)** Density plot showing the distribution of allele frequency. **(E)** Boxplots show the median and interquartile range, with individual data points overlaid. CH^+^ individuals are significantly older than CH^−^ individuals. **(F)** Continuous age–prevalence curves showing the proportion of individuals with CH across age. Curves are stratified by variant allele frequency (VAF) thresholds (≥1% and ≥2%). **(G)** Distribution of patients' age stratified by number of mutations (0, 1, >1). SNVs, single‐nucleotide variants.

A total of 242 FL patients enrolled in the FIL FOLL12 composed the study cohort. The median age was 61.5 years (interquartile range [IQR] 51.7−69.0 years), and 126 (52.1%) were females. A total of 167 (69.3%) patients presented with Ann Arbor Stage IV, and 34 (14.1%) had B symptoms. According to histology, 40 (19.4%) patients were Grade 1, 116 (56.3%) were Grade 2, and 50 (24.3%) were Grade 3A. Based on physician's choice, 141 (58.3%) patients received R‐CHOP and 101 (41.7%) R‐Benda. Complete patient characteristics are reported in Table 1. After a median follow‐up of 98 months, the 8‐year PFS and OS were 54.8% and 86.2%, respectively (Supporting Information S1: Figure 1A,B). Baseline characteristics, including the percentage of patients treated with R‐Benda, were well balanced between patients included in the present molecular analysis and those enrolled in the FOLL12 trial but not analyzed for CH due to unavailable biological material (Supporting Information S8: Table 2). Also, the 8‐year PFS and OS were comparable between the two groups (PFS: HR 1.02, 95% CI 0.80–1.29, P = 0.878; OS: HR 1.02, 95% CI 0.69–1.52, P = 0.908). When stratified by treatment type, HRs were superimposable in both the R‐CHOP group (HR 0.96, 95% CI 0.62–1.49, P = 0.854) and the R‐Benda group (HR 1.03, 95% CI 0.77–1.38, P = 0.852), confirming the representativeness of the studied sub‐cohort also across both treatment groups.

**Table 1:** Patient characteristics.

Characteristic		Value no. (%)
Age (years)	Median	61.5
	Range	51.7–69.0
Gender	Male	116 (47.9)
	Female	126 (52.1)
Ann Arbor stage	II	29 (12.0)
	III	45 (18.7)
	IV	167 (69.3)
B symptoms	No	207 (85.9)
	Yes	34 (14.1)
β2M	≤ULN	117 (48.3)
	>ULN	125 (51.7)
Grading	Grade 1	40 (19.4)
	Grade 2	116 (56.3)
	Grade 3A	50 (24.3)
Hb	≥12 g/dL	203 (83.9)
	<12 g/dL	39 (16.1)
LDH	≤ULN	176 (75.9)
	>ULN	56 (24.1)
Nodal sites	0–4	151 (63.2)
	>4	88 (36.8)
FLIPI	0–1	59 (25.7)
	2	88 (38.3)
	3–5	83 (36.1)
FLIPI2	1–2	146 (60.3)
	3–5	96 (39.7)
Treatment	R‐CHOP	141 (58.3)
	R‐Benda	101 (41.7)

### CH mutational landscape at trial enrollment

A total of 126 mutations were detected in peripheral blood and classified as bona fide CH‐related events (Supporting Information S9: Table 3). Overall, at least one CH mutation was identified in 86 FL patients (35.5%). CH mutations affecting the canonical epigenetic regulators *DNMT3A*, *TET2*, and *ASXL1*, collectively designated as DTA mutations, were detected in 64 (26.4%) patients, including *DNMT3A* in 41 (16.9%), *TET2* in 29 (12.0%), and *ASXL1* in 3 patients (1.2%) (Figure 1A,B). Mutations involving genes of the DDR pathway were less common, being identified in 11 cases and targeting *PPM1D* in 4 patients (1.7%), *TP53* in 3 patients (1.2%), and *CHEK2* and *RAD21* in 2 cases each (0.8%). The distribution of CH mutation types is shown in Figure 1C.

**Figure 2:**
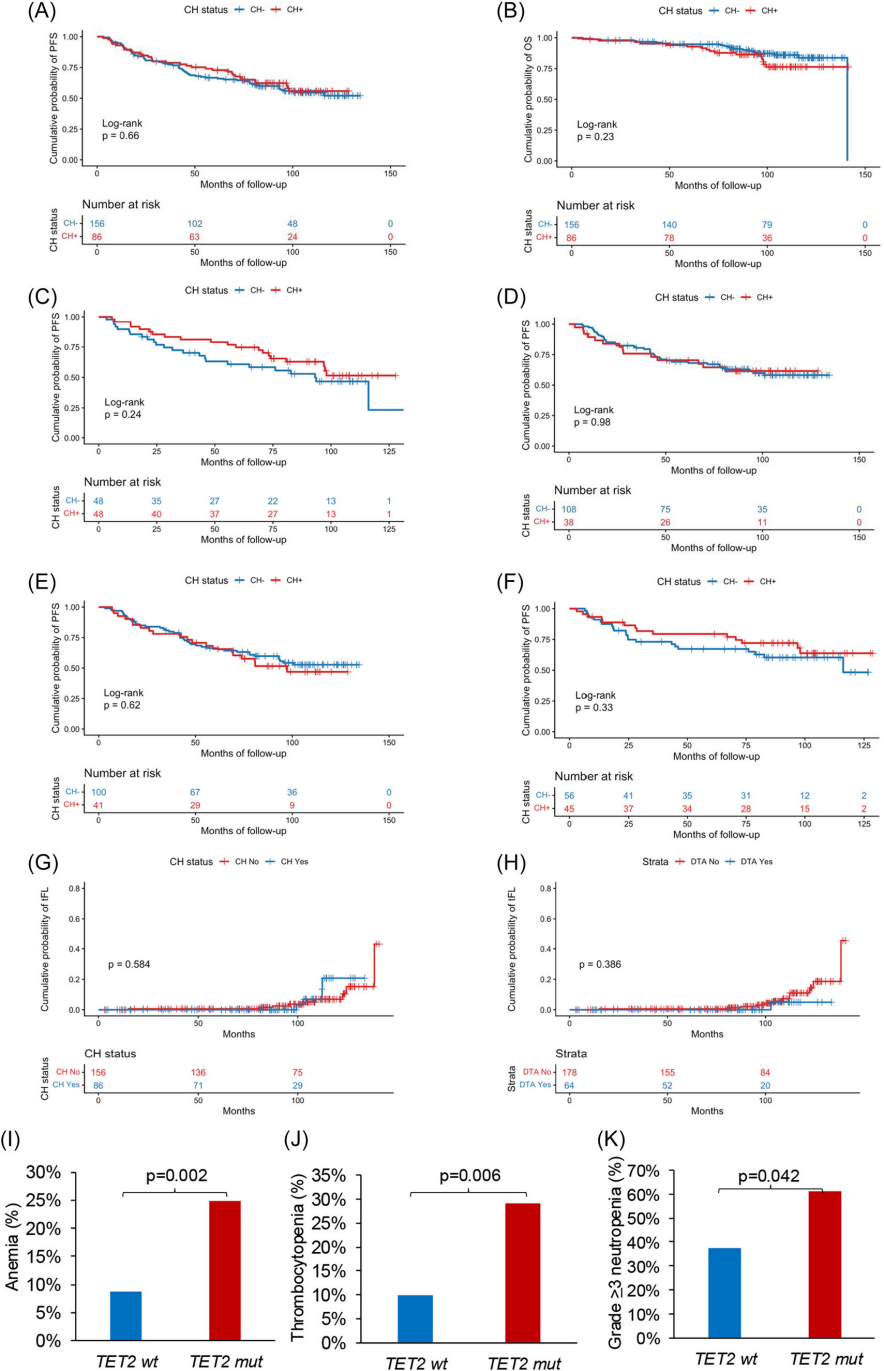
**Clinical impact of clonal hematopoiesis (CH) in follicular lymphoma (FL) patients at trial enrollment.** Kaplan–Meier estimates of **(A)** progression‐free survival (PFS) and **(B)** overall survival (OS) according to the presence of any CH mutation. CH^+^ patients are represented by the red curves, while CH^−^ patients are represented by the blue curves. Kaplan–Meier estimates of PFS in **(C)** patients aged ≥65 years, **(D)** in patients aged <65 years, **(E)** in patients treated with R‐CHOP and **(F)** in patients treated with R‐Benda according to CH status. Kaplan–Meier estimates of the cumulative probability of histologic transformation according to CH **(G)** and DTA **(H)** mutation status. P‐values are displayed adjacent to the curves. Bar charts showing the prevalence of **(I)** anemia, **(J)** thrombocytopenia, and **(K)** Grade ≥3 neutropenia occurring during induction CIT, according to *TET2* mutation status. *TET2*‐mutated patients are represented by red bars, while *TET* wild‐type patients are shown by blue bars. P‐values are displayed above the histograms.

The median VAF of CH mutations at baseline was 1.87% (IQR 1.01–69.08) (Figure 1D and Supporting Information S2: Figure 2A). CH prevalence significantly increased with age reaching 60% among individuals aged ≥75 years (Figure 1E,F). Also, patients carrying multiple CH mutations (12.3% of FL patients) were older (median 68.5 vs. 64.0 years, P = 0.037) compared to patients with a single CH mutation (Figure 1G). No associations were observed between CH mutations and other baseline FL clinical features.

### CH at trial enrollment does not impact on survival outcomes in FL

**Figure 3:**
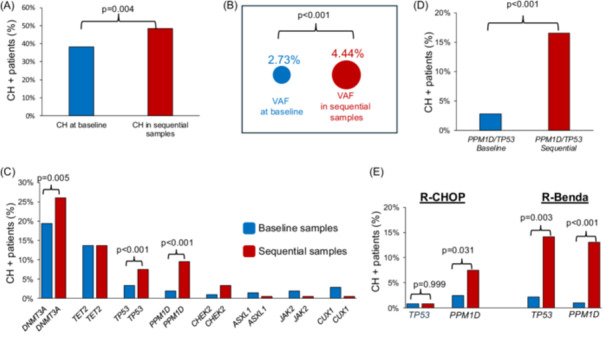
**Impact of chemoimmunotherapy (CIT) on clonal hematopoiesis (CH) prevalence in follicular lymphoma (FL). (A)** CH prevalence in FL significantly increases from baseline (blue) to sequential post‐treatment samples (red). **(B)** Variant allele frequencies (VAFs) of CH mutations detected at baseline (blue) and post‐treatment (red). **(C)** Distribution of CH mutation types at baseline and follow‐up. **(D)** Enrichment of *PPM1D* and *TP53* mutations over time in sequential samples. **(E)** Treatment‐specific expansion of *PPM1D* and *TP53* mutations in patients receiving R‐CHOP or R‐Benda. P‐values are shown above the corresponding comparisons.

CH status, including both overall CH and the canonical DTA subset (*DNMT3A*, *TET2*, and *ASXL1*), was evaluated for its association with PFS, OS, and risk of histologic transformation to aggressive lymphoma. CH^+^ patients exhibited PFS rates comparable to CH⁻ patients, with an 8‐year PFS of 62.3% versus 55.9% (P = 0.660) and an 8‐year OS of 86.4% versus 87.2% (P = 0.230) (Figure 2A,B). Similarly, DTA‐mutated patients showed no evidence of poorer prognosis, with 8‐year PFS and OS estimates mirroring those of DTA wild‐type counterparts (8‐year PFS: 62.8% vs. 56.6%, P = 0.504; 8‐year OS: 85.1% vs. 87.6%, P = 0.527) (Supporting Information S3: Figure 3A,B). Collectively, neither overall CH nor DTA CH mutations demonstrated an adverse impact on clinical outcomes.

Because CH prevalence increases with age, outcomes were also evaluated across age strata. Notably, CH^+^ and CH^−^ patients exhibited superimposable PFS outcomes irrespective of age category. Among individuals >65 years, CH^+^ patients had an 8‐year PFS of 62.9% versus 46.7% in CH^−^ patients (P = 0.240) (Figure 2C). In patients <65 years, CH^+^ individuals showed an 8‐year PFS of 61.6% compared with 59.9% for CH^−^ patients (P = 0.980) (Figure 2D). These findings indicate that the clinical neutrality of CH at trial enrollment extends across age‐dependent risk groups.

Also, CH does not impact on PFS when subdividing patients according to induction therapy received, namely R‐CHOP and R‐Benda. Among R‐CHOP treated patients, CH^+^ cases had an 8‐year PFS of 54.4% versus 51.6% in CH^−^ patients (P = 0.618) (Figure 2E). Among R‐Benda‐treated patients, CH^+^ cases showed an 8‐year PFS of 60.4% compared with 68.1% for CH^−^ patients (P = 0.333) (Figure 2F).

In this cohort, 13/242 patients (5.4%) experienced histologic transformation to aggressive lymphoma. The presence of CH was not associated with an increased risk of transformation (hazard ratio [HR] 1.40, P = 0.584). Transformation rates were comparable between CH^+^ and CH^−^ patients (CH^+^: 4.7%; CH^−^: 5.8%) (Figure 2G), and no enrichment of DTA mutations was observed among transformed cases (HR 0.40, P = 0.386) (Figure 2H).

Collectively, these findings provide reassurance that CH mutations before treatment do not meaningfully alter the biological trajectory of FL nor predispose to histologic transformation.

### CH predisposes to specific types of therapy‐related toxicities during induction CIT

The risk of therapy‐related hematological toxicities during induction CIT was evaluated in relation to CH and across recurrent CH‐related gene mutations. No significant differences in hematological toxicity rates were observed between CH^+^ and CH^−^ cases (Supporting Information S10: Table 4). However, *TET2* mutations were significantly associated with a higher prevalence of anemia, thrombocytopenia, and Grade ≥3 neutropenia. In detail, anemia was present in 41.4% (*N* = 12) of *TET2*‐mutated patients compared to 16.9% (*N* = 36) of *TET2* wild‐type patients (P = 0.002) (Figure 2I), and thrombocytopenia was present in 24.1% (*N* = 7) of *TET2*‐mutated patients compared to 8.0% (*N* = 17) of *TET2* wild‐type patients (P = 0.006) (Figure 2J). Also, *TET2* mutations were significantly associated with Grade ≥3 neutropenia, occurring in 55.2% (*N* = 16) *TET2*‐mutated patients compared to 35.7% (*N* = 76) wild‐type patients (P = 0.042) (Figure 2K).

### CIT drives selective expansion of CH prevalence

To evaluate CH dynamics after the genotoxic stimuli imposed by CIT, 211 patients provided with one or more sequential samples were analyzed. The median time between baseline and sequential samples was 30 months (IQR 12–36 months). The complete list of gene mutations identified in sequential samples is reported in Supporting Information S11: Table 5 and Supporting Information S2: Figure 2B–F).

Overall, CIT significantly expanded both the prevalence and the VAF of CH clones. The total number of CH mutations increased from 116 to 180 (P = 3.22 × 10⁻⁷), which was reflected by an increase in CH^+^ patients from 37.4% (*N* = 79) to 47.4% (*N* = 100) in post‐treatment samples (P = 0.004) (Figure 3A). Also, CIT significantly expanded the median VAF of detectable pre‐existing clones from 2.7% at baseline to 4.4% in post‐CIT samples (P < 0.001) (Figure 3B).

Evaluation of treatment‐associated shifts in the patient‐level prevalence of CH mutations revealed that CIT profoundly reshaped the CH landscape, driving a significant expansion in the proportion of patients harboring *DNMT3A*, *TP53*, and *PPM1D* mutations (Figure 3C). Overall, the prevalence of *DNMT3A*‐mutated patients increased from 18.4% (*N* = 39) at baseline to 26.5% (*N* = 56) post‐CIT (P = 0.005), of *TP53*‐mutated patients from 0.9% (*N* = 2) to 6.6% (*N* = 14) (P < 0.001), and of *PPM1D*‐mutated patients from 1.9% (*N* = 4) to 9.9% (*N* = 21) (P < 0.001). Consequently, the prevalence of patients with CH mutations of DDR genes, namely *TP53* and/or *PPM1D*, expanded from 2.8% (*N* = 6) to 16.6% (*N* = 35) (P < 0.001), with near‐complete mutual exclusivity of the two CH mutations (Figure 3D). Only one patient acquired both lesions.

By leveraging the distinct cytotoxic pressures of R‐CHOP and R‐Benda, regimen‐specific selection patterns of CH were unveiled. Most notably, the prevalence of *TP53*‐mutated patients underwent a significant and exclusive expansion after R‐Benda, rising from 0.9% (*N* = 1) at baseline to 15.0% (*N* = 12) post‐CIT (P < 0.001), while remaining completely unchanged after R‐CHOP (0.9%–0.9%, P = 0.999) (Figure 3E). Importantly, all newly detected *TP53* mutations occurred in MRD‐negative patients, confirming their origin from CH rather than from residual FL cells. In contrast, the prevalence of *PPM1D*‐mutated patients increased under both regimens, from 2.5% to 7.6% with R‐CHOP (P = 0.031) and from 1.1% to 11.9% with R‐Benda (P < 0.001), whereas the prevalence increase of *DNMT3A*‐mutated patients was preferentially associated with R‐CHOP (15.0% to 25.2%, P = 0.004) (Figure 3E).

### Clonal fitness of CH differs under R‐CHOP versus R‐Benda

**Figure 4:**
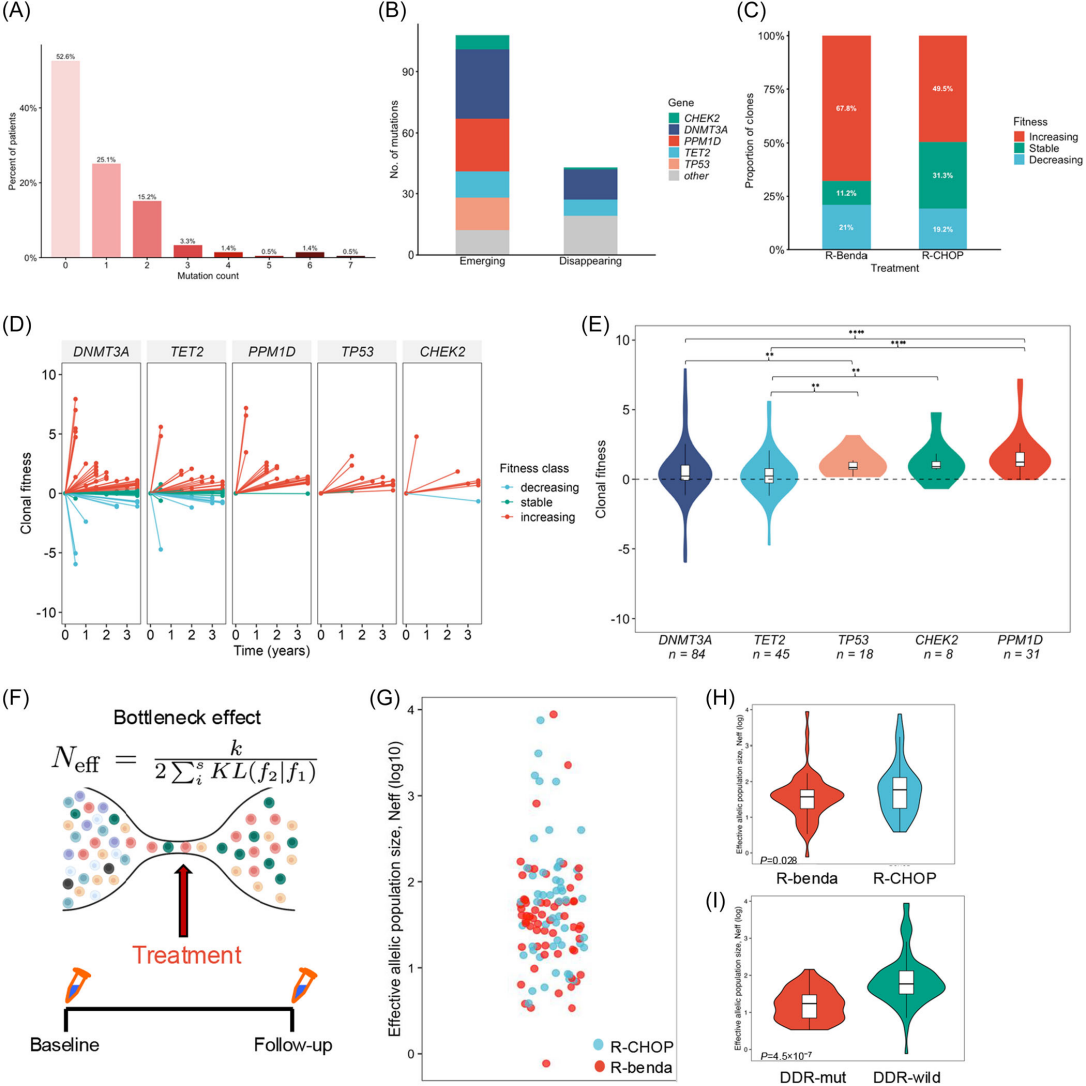
**Longitudinal dynamics, fitness, and clonal structure of clonal hematopoiesis (CH) under chemoimmunotherapy (CIT). (A)** Percentage of follicular lymphoma (FL) patients with and without CH stratified by the number of mutations (0–7) in sequential samples after treatment. **(B)** Comparison of emerging and disappearing CH mutations by gene. **(C)** Proportion of clones showing increasing, decreasing, or stable dynamics following treatment with R‐Benda or R‐CHOP. **(D)** Gene‐specific trajectories of CH clonal fitness over time, showing increasing, stable, and decreasing clones across major CH‐associated genes post‐treatment. **(E)** Distribution of clonal fitness across major CH‐associated genes, showing gene‐specific differences in median fitness and variability (*P < 0.05; **P < 0.01; ***P < 0.001; and ****P < 0.0001). **(F)** Schematic illustration of the treatment‐induced bottleneck effect, which, by exerting a selective pressure, reduces clonal diversity of CH and reshapes the effective population size. **(G)** Effective allelic population size of CH (*N*_eff_, log10 scale) across samples, highlighting variability in clonal population dynamics. Comparison of effective allelic population size of CH (*N*_eff_, log10 scale) **(H)** between patients treated with R‐Benda and R‐CHOP and **(I)** between DNA damage response (DDR)‐mutant and DDR wild‐type CH clones.

Clonal fitness, defined as the annual change of VAF as a logistic growth, was inferred to better investigate the clonal dynamics of CH. Clones were classified as increasing, stable, or decreasing as specified in the Materials and Methods section. Among all clones detected at baseline or post‐CIT, 128 (57.1%) exhibited increasing VAFs over time, whereas 49 (21.9%) decreased and 47 (21.0%) remained stable (Figure 4A–C). Among recurrent mutations, DDR‐associated variants (*PPM1D*, *CHEK2*, and *TP53)* showed the highest median fitness values (1.232, 0.908, and 0.833, respectively), significantly exceeding those of DTA‐associated variants, particularly *DNMT3A* (0.229) and *TET2* (0.233) (Figure 4D). Gene‐wise comparisons reinforced this pattern, demonstrating significantly greater fitness among DDR‐mutated clones, with *PPM1D* exhibiting the strongest growth advantage (Figure 4E).

Treatment‐specific patterns were also evident, corroborating that CH mutations post‐CIT follow different expansion pathways according to treatment. Overall, R‐Benda was associated with a significantly higher proportion of CH clones with increasing VAF compared to R‐CHOP (79/125 vs. 49/100, P = 0.042), suggesting a stronger selective advantage for specific mutations under this regimen. Both treatments promoted the expansion of CH clones, although with a different pattern. Notably, increased fitness dynamics of *TP53*‐mutated CH clones (n = 17) was observed exclusively following R‐Benda therapy. Conversely, both regimens promoted the clonal fitness of *PPM1D* mutations (R‐Benda *n* = 20; R‐CHOP *n* = 10). Although *DNMT3A* mutations were more common in R‐CHOP patients, post‐treatment clonal dynamics were similar across regimens, with comparable proportions of increasing, stable, and decreasing clones.

### Clonal bottlenecks and longitudinal stability of CH architecture

To quantify patient‐level changes in CH clonal architecture under R‐CHOP and R‐Benda, we estimated an effective allelic population size ($N_{\text{eff}}$) for each patient based on pre‐ and post‐treatment VAF distributions (Figure 4F). Lower $N_{\text{eff}}$ values indicate a stronger treatment bottleneck with emerging clonal dominance, whereas higher values reflect weaker selective pressure and preserved clonal diversity.

R‐CHOP was associated with higher $N_{\text{eff}}$ values (median 58.9; range 3.9–7828.4), indicating relatively weaker treatment bottlenecks and preservation of broad clonal diversity. In contrast, R‐Benda produced markedly lower $N_{\text{eff}}$ values (median 37.5; range: 0.8–8791.5; P = 0.028), reflecting stronger selective pressure and more pronounced contraction of the clonal pool (Figure 4G,H). This difference suggests that R‐Benda exerts a higher impact on clonal architecture, creating a tighter therapeutic bottleneck that may facilitate the emergence of fitter or therapy‐resistant clones. Notably, patients harboring DDR mutations exhibited significantly lower $N_{\text{eff}}$ values compared with those without DDR alterations (median $N_{\text{eff}}$ 16.7 vs. 59.6, P = 4.5 × 10⁻7), consistent with heightened treatment‐driven clonal restriction in this subgroup (Figure 4I).

To further refine the reconstruction of CH clonal trajectories, we additionally analyzed an intermediate timepoint in 75 CH^+^ patients (median time from CIT: 12 months). VAFs were highly concordant between the post‐CIT intermediate and final timepoint samples (Pearson *r* = 0.91, P < 1 × 10⁻^60^) (Supporting Information S4: Figure 4A,B). The median VAF at the intermediate timepoint was 2.4%, compared with 2.3% at the last timepoint, reflecting minimal overall change in clonal abundance during this interval. Notably, fitness estimates were higher in the earlier interval (baseline to T1) compared to the later interval (T1 to T2) (median fitness 0.34 vs. 0.00; P < 0.001) (Supporting Information S4: Figure 4C,D). These findings indicate that, in most cases, clonal dynamics observed at final follow‐up was already established early after treatment, with CH clones after CIT generally following persistent trajectories rather than fluctuating patterns.

### Clonal fitness of CH has a detrimental effect on FL survival

**Figure 5:**
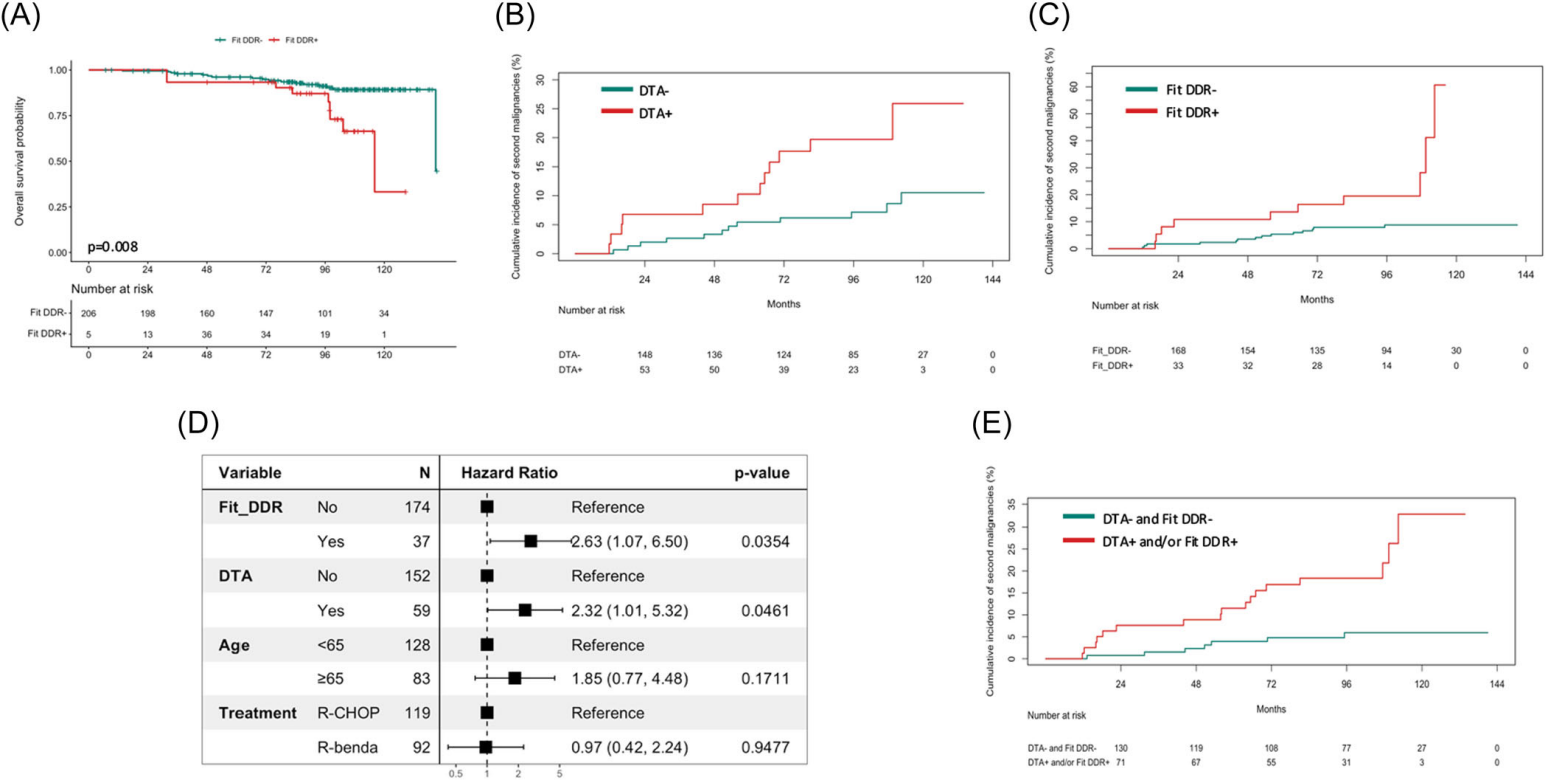
**Impact of baseline DTA mutations and post‐chemoimmunotherapy (CIT) fit DNA damage response (DDR) clonal expansion of clonal hematopoiesis (CH) on the risk of second primary malignancies in follicular lymphoma (FL). (A)** Simon–Makuch estimates of overall survival (OS) according to the presence of fit DDR clones. Fit DDR^+^ patients are represented by the red curves, while fit DDR^−^ patients are represented by the green curves. Because the clone status was modeled as a time‐dependent covariate, patients could transition between groups over time; accordingly, numbers at risk may fluctuate at later timepoints. **(B)** Cumulative incidence of second primary malignancies according to baseline DTA mutation status (DTA^+^ vs. DTA^−^). Death was treated as a competing risk. **(C)** Cumulative incidence of second primary malignancies according to the presence of post‐treatment fit DDR mutations (fit DDR^+^ vs. fit DDR^−^). Death was treated as a competing risk. **(D)** Multivariable Cox regression analysis for second primary malignancies, after adjusting for baseline DTA status, post‐CIT fit DDR status, age, and treatment regimen. **(E)** Integrated cumulative incidence of second primary malignancies according to baseline DTA mutations and/or fit DDR clones (DTA and/or fit DDR clones vs. neither). Death was treated as a competing risk.

Although baseline CH, including baseline DTA mutations, was not associated with inferior OS per se (Supporting Information S3: Figure 3B), the post‐treatment emergence of fit DDR clones (clones showing increasing VAF according to the fitness model) identified a clinically distinct subgroup of FL patients with markedly reduced survival. Using a time‐dependent Cox model, patients who developed fit DDR clones experienced a significantly shorter OS compared with those who did not (HR 3.28, 95% CI 1.37–7.86, P = 0.008) (Figure 5A). Importantly, incorporating baseline DTA mutations into a combined model with fit DDR status did not neutralize the adverse prognostic effect of post‐CIT DDR clonality; rather, the combined profile remained significantly associated with inferior OS (HR 2.85, 95% CI 1.26–6.45, P = 0.012) (Supporting Information S5: Figure 5A).

### DTA mutations and fit DDR clones predict second primary malignancies in FL

The long‐term follow‐up of the FOLL12 trial enables the integration of molecular findings with late toxicities, particularly the development of second primary malignancies. At a median follow‐up of 98 months (range, 3.3–141.4), 28 patients developed a second primary malignancy (Supporting Information S12: Table 6), corresponding to a 9‐year cumulative incidence of 11.9% (95% CI, 6.3–15.1).

At baseline, the 9‐year cumulative incidence of second primary malignancies was significantly higher in patients harboring DTA mutations compared with DTA wild‐type patients (21.3% vs. 7.3%; P = 0.0017) (Figure 5B). Consistently, multivariable Cox regression analysis corroborated baseline DTA mutations as an independent predictor of second primary malignancies after adjusting for age and treatment regimen (HR 2.66, 95% CI 1.35–5.67, P = 0.011) (Supporting Information S5: Figure 5B). Patients experiencing progression and receiving second‐line therapy for FL did not have an increased risk of second primary malignancies (HR 0.25, 95% CI 0.06–1.07; P = 0.062).

The presence of DDR mutations at baseline was not associated with an increased risk of second primary malignancies (P = 0.528) (Supporting Information S5: Figure 5C). Conversely, after treatment, patients with fit DDR mutations exhibited a markedly increased 9‐year cumulative incidence of second primary malignancies, being 28.2% compared with 8.8% in cases without fit DDR mutations (P < 0.001) (Figure 5C). The association of fit DDR mutations with second primary malignancies was then confirmed in a multivariable analysis, adjusting for baseline DTA status, age, and treatment regimen (HR 2.63, 95% 1.07–6.05, P = 0.035) (Figure 5D).

Since both baseline DTA and post‐treatment fit DDR mutations individually and independently increased the risk of second primary malignancies, we next evaluated their integrated effect. Patients harboring DTA and/or fit DDR mutations demonstrated a significantly higher cumulative incidence compared to those lacking both events (21.8% vs. 5.9% at 9 years), rising to 32.7% at 10 years (P < 0.001) (Figure 5E). This predictive model for second primary malignancies remained statistically significant also after adjusting for age and treatment regimen (HR 4.12, 95% CI 1.62–10.44; P = 0.003) (Supporting Information S5: Figure 5D), underscoring the robust and complementary prognostic impact of these two biologically distinct clonal events.

## DISCUSSION

In this study, we provide the first comprehensive evaluation of myeloid CH in patients with advanced‐stage FL requiring frontline therapy, leveraging the prospective Phase III FOLL12 trial of the Fondazione Italiana Linfomi. Baseline CH does not adversely affect treatment outcomes with either R‐CHOP or R‐Benda; however, it is associated with increased hematologic toxicity during treatment. CIT exerts substantial genotoxic pressure on hematopoietic clones, resulting in distinct bottlenecks of clonal selection that differ under R‐CHOP versus R‐Benda. The combination of baseline DTA‐mutated CH and post‐CIT fit DDR clones enhances the risk of second primary malignancies, ultimately contributing to inferior long‐term clinical outcomes.

CH analysis was performed on whole peripheral blood, consistent with approaches used to assess CH in the general population without an overt lymphoma diagnosis.^5^, ^6^ In some indolent B‐cell neoplasms, however, peripheral blood can be contaminated by circulating leukemic cells, potentially affecting the sensitivity and accuracy of CH detection, particularly when mutations may contribute to both CH and the underlying malignancy.^34^, ^35^ In FL, by contrast, common CH‐associated mutations are generally not implicated in lymphoma pathogenesis, and *TP53* mutations are rarely observed at diagnosis. Furthermore, the availability of MRD data in the FOLL12 trial enabled us to assess the presence or absence of FL cells in the peripheral blood. Importantly, all cases harboring detectable *TP53* variants were MRD‐negative, supporting a CH origin for all identified *TP53* mutations.

Across B‐cell malignancies, prior studies have reported heterogeneous associations between CH and clinical outcomes, with the strongest adverse signals observed in patients undergoing stem‐cell transplantation.^21^, ^24^, ^35^, ^36^, ^37^, ^38^, ^39^ The present study provides evidence that baseline CH mutations do not influence outcomes of advanced‐stage FL. PFS and OS were comparable in patients with or without CH, independent of age or frontline regimen (R‐CHOP or R‐Benda). Also, CH did not increase the risk of histologic transformation from FL to DLBCL.

CH *TET2* mutations were associated with increased hematologic toxicity during induction CIT, including anemia, thrombocytopenia, and neutropenia. This pattern is biologically plausible: *TET2* loss impairs erythropoiesis through altered SCF/c‐KIT signaling and defective CFU‐E maturation,^40^ and it produces neutrophils with immature transcriptional profiles, reflecting broader myeloid dysfunction.^41^, ^42^ Also, *TET2* deficiency leads to dysregulated megakaryocyte maturation and platelet production.^43^, ^44^ Together, these defects may reduce the resilience of *TET2*‐mutant progenitors across lineages, providing a biologic rationale for the higher rates of hematologic toxicity observed in *TET2*‐mutant FL patients receiving CIT.

Our longitudinal analyses indicate that CIT acts as a potent evolutionary pressure on the hematopoietic compartment, selectively favoring CH clones with superior biological fitness. More than half of all detectable mutations increased in VAF over time post CIT, with DDR‐associated lesions consistently exhibiting the highest fitness and emerging as dominant clones following treatment. Regimen‐specific effects further refined this pattern. R‐Benda imposed a more pronounced treatment bottleneck than R‐CHOP and uniquely promoted the expansion of highly fit *TP53*‐mutated clones. This observation may be ascribed to the dual mechanism of action of bendamustine as both an alkylating agent and a purine analog.^45^ The combination of extensive DNA crosslinking and replication stress may preferentially advantage p53‐deficient progenitors that are less constrained by p53‐mediated checkpoint responses.^46^ In addition, bendamustine is associated with deeper and more prolonged immunosuppression, raising the possibility that diminished immune surveillance may contribute to reduced clearance of *TP53*‐mutant hematopoietic cells, thereby facilitating their expansion.^47^ Notably, the results of the FOLL12 trial show that fitness‐driven trajectories were established early and remained stable over time, suggesting that CIT rapidly determines the post‐treatment evolutionary course of CH.^26^ Together, these findings position DDR‐mutated clones as a central determinant of CH dynamics following frontline CIT.

Lymphoma patients, particularly those treated with CIT, show an increased incidence of second primary malignancies, yet reliable predictors are still lacking.^48^ The FOLL12 platform includes long‐term clinical follow‐up of up to 10 years after trial enrollment, providing a unique opportunity to identify biomarkers associated with the development of second malignancies.^49^ This study demonstrates that both baseline DTA CH mutations and the presence of fit DDR CH clones after CIT are associated with a higher risk of second primary malignancies, independent of age and treatment regimen. Most second malignancies observed were solid tumors, suggesting a potential role for CH in promoting susceptibility to secondary cancers, likely through inflammatory pathways and interactions within the tumor microenvironment.^25^, ^50^, ^51^, ^52^, ^53^

As previously stated, our study focused on myeloid CH analysis. Lymphoid‐CH–related genes were not included in our targeted panel because all patients in this study already had a diagnosis of lymphoma, which represents the clinical condition that lymphoid‐CH is thought to predispose to.^30^ Consequently, the detection of variants in lymphoid‐associated genes in peripheral blood would not allow us to reliably distinguish whether these mutations originated from circulating normal lymphoid cells or from lymphoma cells. In addition, the design of the FIL FOLL12 clinical trial did not include the collection of viable frozen samples that enable the dissection of different peripheral blood cell compartments. The issue of CH compartments falls beyond the scope of this study and deserves to be evaluated in cohorts provided with frozen viable cells specifically collected for this purpose.

Our analysis presents some limitations. First, CH was assessed on whole peripheral blood, which may underestimate the contribution of rare or compartmentalized clones, although the availability of MRD data mitigated concerns about contamination by residual FL cells.^54^ Second, although the association between CH and second primary malignancies is compelling, mechanistic links cannot be definitively established from observational data. Lastly, evaluating CH in FL patients receiving emerging chemo‐free regimens will clarify whether novel approaches may overcome the detrimental effects of CIT on long‐term outcomes due to CH presence and expansion.

Overall, CH profiling emerges as a novel and valuable biomarker in FL, also capable of predicting long‐term toxicities that are key endpoints in indolent lymphoid malignancies characterized by long‐lasting survival. Considering the potential contribution of CH to responses and therapy‐related toxicities to T‐cell engager therapies in lymphoid malignancies,^55^, ^56^, ^57^ the design of a dedicated clinical trial using CH as a predictive biomarker for both therapeutic efficacy and treatment‐related toxicities represents a next step to validate the role of CH in the evolving treatment landscape of FL.

## AUTHOR CONTRIBUTIONS

**Nawar Maher**: Conceptualization; methodology; data curation; writing—original draft; writing—review and editing. **Riccardo Moia**: Conceptualization; writing—original draft; funding acquisition; methodology; writing—review and editing; supervision; data curation; software; project administration. **Mohammad Almasri**: Methodology; software; formal analysis; data curation; writing—review and editing. **Luca Cividini**: Methodology; data curation; formal analysis; writing—review and editing. **Elisa Genuardi**: Methodology; writing—review and editing. **Chiara Cosentino**: Methodology; software; writing—review and editing; data curation. **Roberta Soscia**: Methodology; writing—review and editing. **Giovanni Manfredi Assanto**: Methodology; writing—review and editing. **Rita Tavarozzi**: Methodology; writing—review and editing. **Maria Carmela Vegliante**: Methodology; writing—review and editing. **Luisa Lorenzi**: Methodology; writing—review and editing. **Annalisa Andorno**: Methodology; writing—review and editing. **Luca Arcaini**: Methodology; writing—review and editing. **Simone Ragaini**: Methodology; writing—review and editing. **Benedetta Puccini**: Methodology; writing—review and editing. **Caterina Patti**: Methodology; writing—review and editing. **Armando Santoro**: Methodology; writing—review and editing. **Gloria Margiotta‐Casaluci**: Methodology; writing—review and editing. **Vittorio Ruggero Zilioli**: Methodology; Writing—review and editing. **Manuela Zanni**: Methodology; writing—review and editing. **Sonia Ronconi**: Methodology; writing—review and editing. **Francesco Di Raimondo**: Methodology; writing—review and editing. **Annalisa Arcari**: Methodology; writing—review and editing. **Catello Califano**: Methodology; writing—review and editing. **Claudia Castellino**: Methodology; writing—review and editing. **Annarita Conconi**: Methodology; writing—review and editing. **Tommasina Perrone**: Methodology; writing—review and editing. **Donato Mannina**: Methodology; writing—review and editing. **Caterina Plenteda**: Methodology; writing—review and editing. **Francesco Alesiani**: Methodology; writing—review and editing. **Francesca Gaia Rossi**: Methodology; writing—review and editing. **Angelo Michele Carella**: Methodology; writing—review and editing. **Luigi Marcheselli**: Methodology; writing—review and editing; data curation; formal analysis. **Sara Galimberti**: Methodology; writing—review and editing. **Sabino Ciavarella**: Methodology; writing—review and editing. **Riccardo Bomben**: Methodology; writing—review and editing; funding acquisition. **Ilaria Del Giudice**: Methodology; writing—review and editing; data curation; supervision; funding acquisition. **Marco Ladetto**: Conceptualization; data curation; supervision; writing—review and editing; funding acquisition. **Gianluca Gaidano**: Methodology; conceptualization; writing—review and editing; supervision; data curation; writing—original draft; funding acquisition. **Stefano Luminari**: Methodology; data curation; supervision; writing—review and editing; writing—original draft; funding acquisition. **Simone Ferrero**: Writing—original draft; writing—review and editing; methodology; data curation; supervision; funding acquisition.

## CONFLICT OF INTEREST STATEMENT

R.M. is on the advisory board and received speaker's honoraria from Johnson & Johnson, AbbVie, AstraZeneca, and BeOne. S.F. is a consultant for Johnson & Johnson, EUSA Pharma, AbbVie, and Sandoz, is on the advisory board of Johnson & Johnson, EUSA Pharma, Recordati, Incyte, Roche, AstraZeneca, Italfarmaco, and Behring, received speaker's honoraria from Janssen, EUSA Pharma, Recordati, Lilly, BeOne, Gilead, and Gentili, and received research funding from Gilead and Morphosys. S.L. is on the advisory board of Roche, Novartis, BMS, Kite, BeOne, Incyte, AbbVie, and Regeneron, received speaker's honoraria from Roche, Incyte, AbbVie, BMS, and Kite, and received research and travel grants from Roche and BeOne. Si.R. received speaker's honoraria from Roche, BeOne, Pierre Fabre, and Novartis and received travel grants from Kyte/Gilead. V.R.Z. is on the advisory board and speaker's bureau of AbbVie, AstraZeneca, Kite/Gilead, Novartis, Roche, Sobi, Takeda, Incyte, Janssen, and Lilly, is a consultant of Roche, and received research support from Roche and travel grants from AbbVie, BeOne, Janssen, Lilly, Roche, and Takeda. M.L. has relationships in terms of consultancy, participation in advisory boards, invitation to scientific meetings, institutional research support, and contracts with AbbVie, Acerta, Amgen, ADC Therapeutics, BeOne, Celgene/BMS, Eusapharma, GSKI, Gentili, Gilead/Kite, Novartis, Incyte, Johnson & Johnson, Jazz, Lilly, Regeneron, Roche, and Sandoz, and he has non‐financial interests as PI or strategic investigator in studies supported by Celgene, Johnson & Johnson, BeiGene, ADC Therapeutics. IDG has relationships in terms of participation in advisory boards and consultancy with Roche, AstraZeneca, Janssen, BeOne, and Takeda. A.S. is on the advisory board of BMS, Servier, Gilead, Pfizer, Eisai, Bayer, and MSD, is a consultant of Sanofi and Incyte, and received speaker's honoraria from Takeda, BMS, Roche, AbbVie, Amgen, Celgene, Servier, Gilead, AstraZeneca, Pfizer, Lilly, Sandoz, Eisai, Novartis, Bayer, and MDS. G.G. received honoraria from AbbVie, AstraZeneca, BeOne, Hikma, Incyte, Johnson & Johnson, and Lilly. L.A. received speaker's honoraria from AstraZeneca, Novartis, Kite/Gilead, Beigene, and AbbVie, is on the advisory board of Roche, Janssen‐Cilag, Incyte, EUSA Pharma, Celgene/Bristol Myers Squibb, Kite/Gilead, Novartis, and BMS, and received support for attending meetings and/or travel from Roche and AstraZeneca.

## FUNDING

This work was supported by “Bando Giovani Ricercatori 2022” of the Fondazione Italiana Linfomi founded by Fondazione GRADE Onlus e Fondazione Maramotti Linfomi; Molecular bases of disease dissemination in lymphoid malignancies to optimize curative therapeutic strategies (AIRC5 x 1000 No. 21198), Associazione Italiana per la Ricerca sul Cancro (AIRC) Foundation Milan, Italy; the AGING Project – Department of Excellence – DIMET, Università del Piemonte Orientale, Novara, Italy; AIL Novara VCO ODV, Novara, Italy; Progetto di Ricerca Sanitaria Finalizzata 2021 (RF‐2021‐12371972, CUP G13C21001540001), Torino, Italy and Alessandria, Italy; Young Researcher Award 2018 by Fondazione Italiana Linfomi (FIL), Fondazione Giulia Maramotti and Fondazione Grade ONLUS, Reggio Emilia, Italy; Bando “FIL CLUB 2021” by Fondazione Italiana Linfomi (FIL), Alessandria and Modena, Italy; and Fondi di Ricerca Locale, Università di Torino, Italy. Open access publishing facilitated by Universita degli Studi del Piemonte Orientale Amedeo Avogadro, as part of the Wiley ‐ CRUI‐CARE agreement.

## Supporting information

Supporting Information.

Supporting Information.

Supporting Information.

Supporting Information.

Supporting Information.

Supporting Information.

Supporting Information.

Supporting Information.

Supporting Information.

Supporting Information.

Supporting Information.

Supporting Information.

## Data Availability

The data that support the findings of this study are available from the corresponding author upon reasonable request.
