# Supplementary figures and images for: Clonal hematopoiesis dynamics influences long‐term outcomes of follicular lymphoma: Results from FIL FOLL12 trial
Source: Hemasphere. 2026 May 20;10(5):e70393. doi: 10.1002/hem3.70393 (PMC13240525; doi:10.1002/hem3.70393)

**A**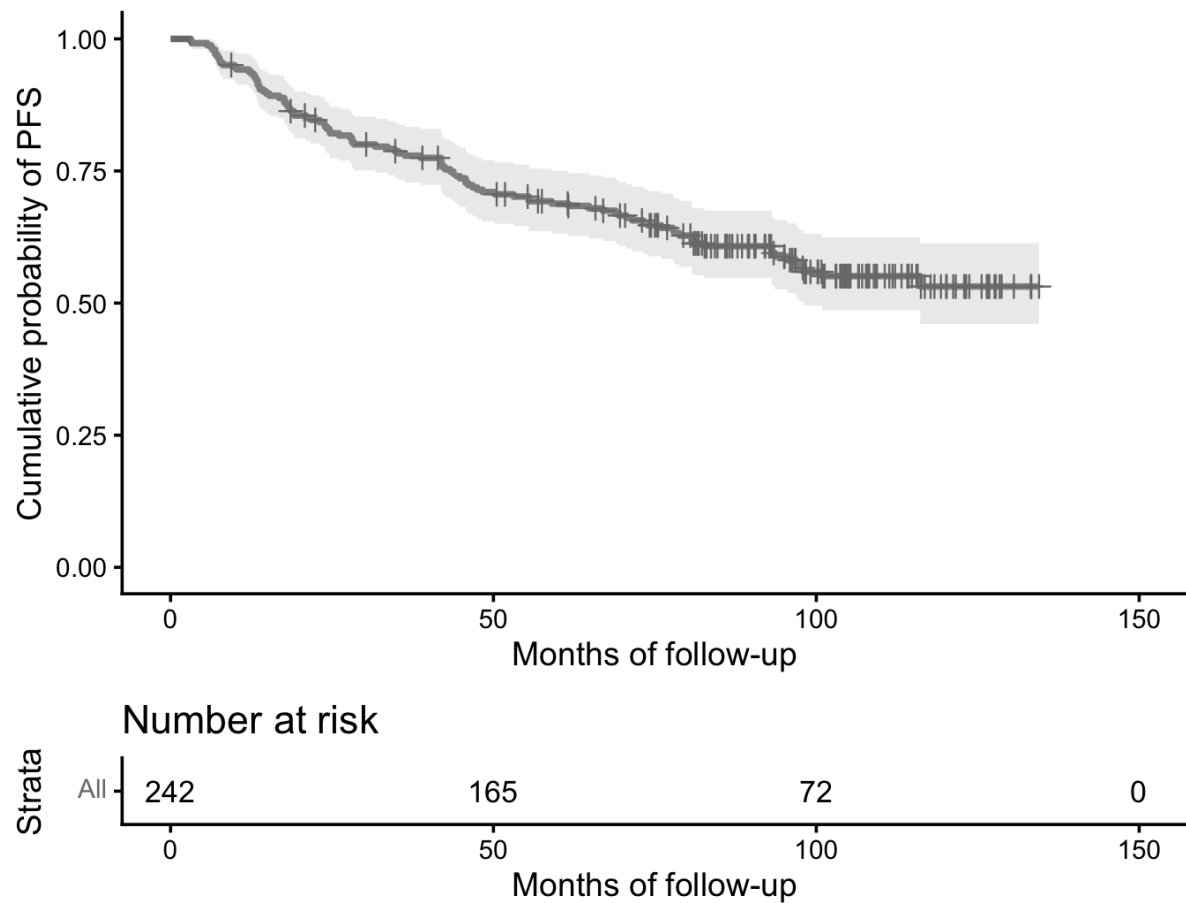**B**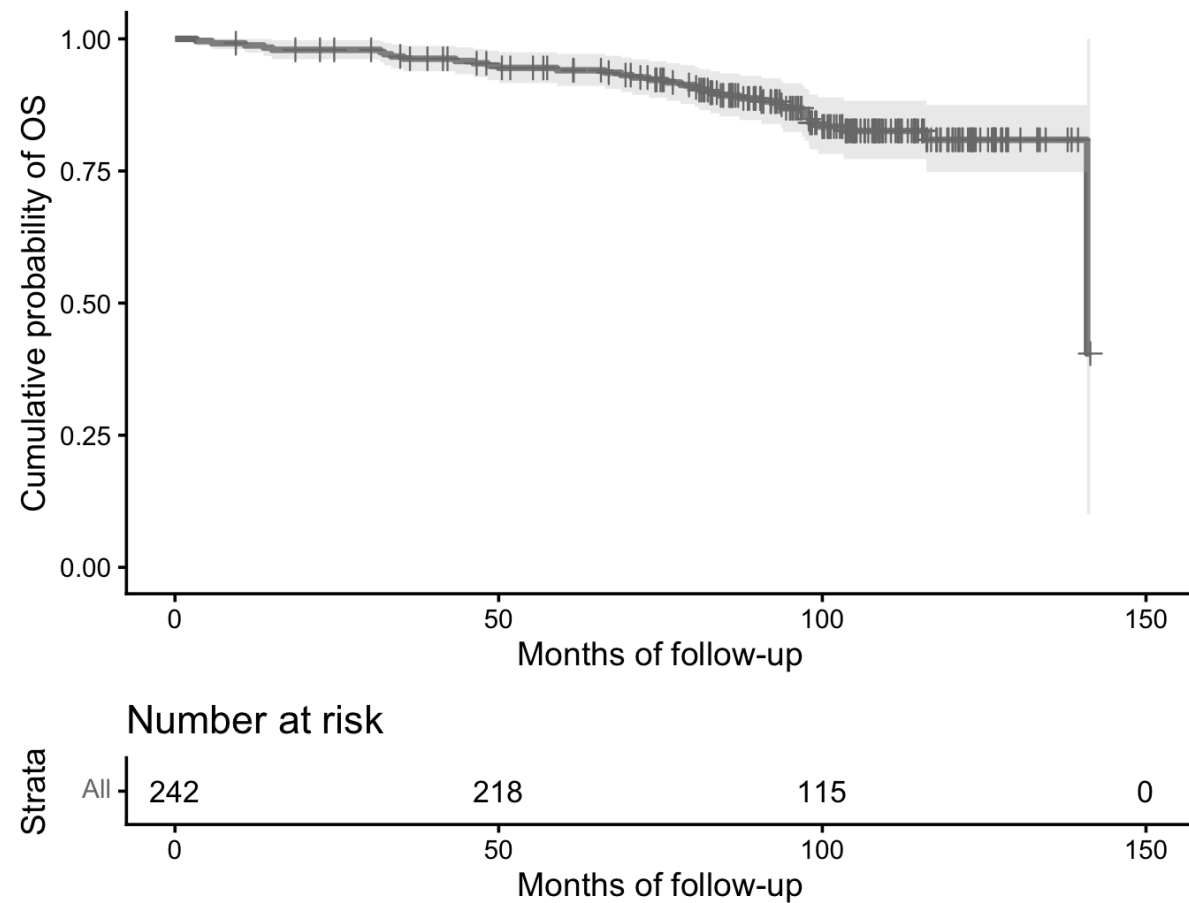

Figure S1

Supplement: Supplementary file 1 — Supporting Information. [file HEM3-10-e70393-s011.pdf]

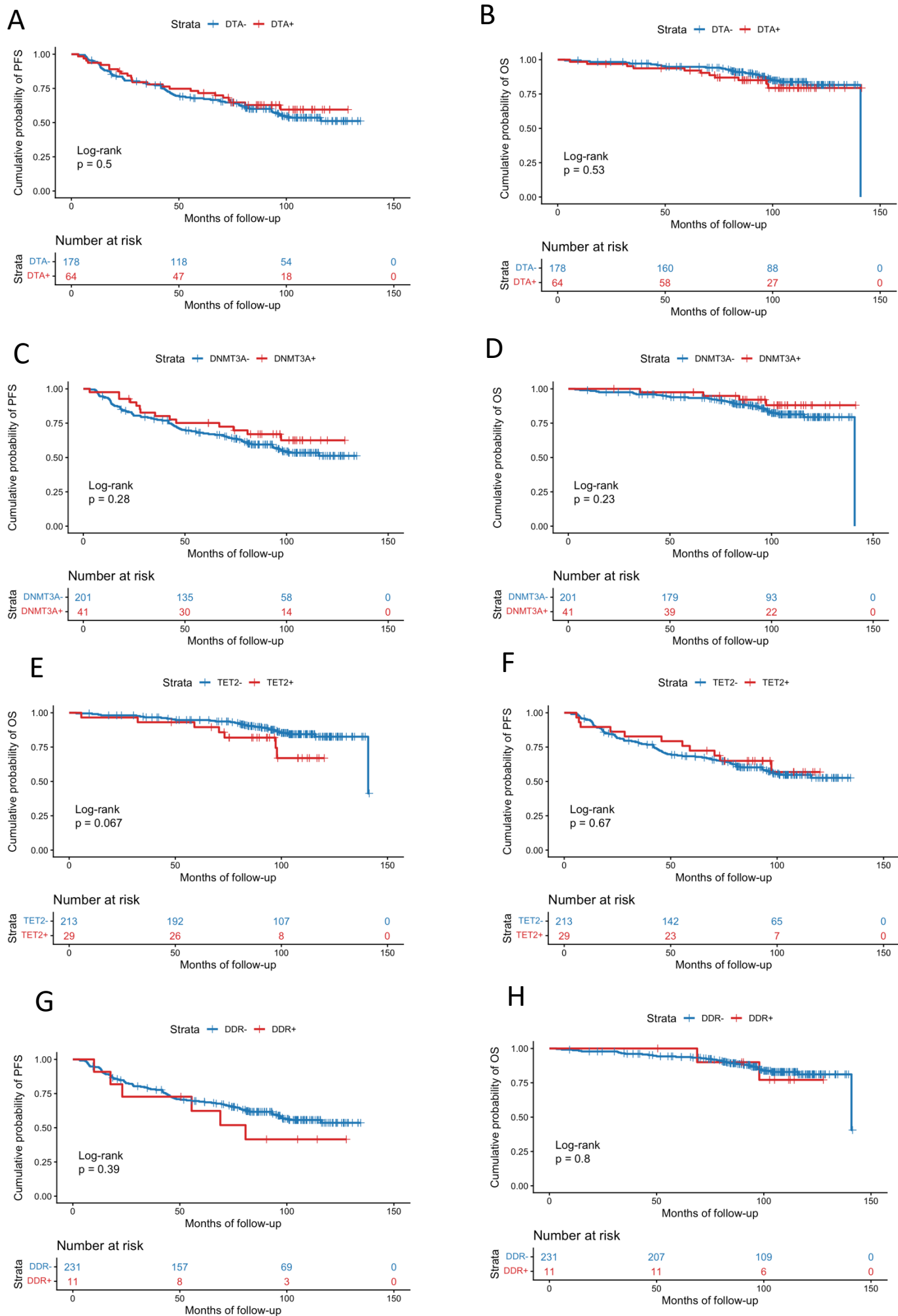

Figure S3

Supplement: Supplementary file 3 — Supporting Information. [file HEM3-10-e70393-s003.pdf]

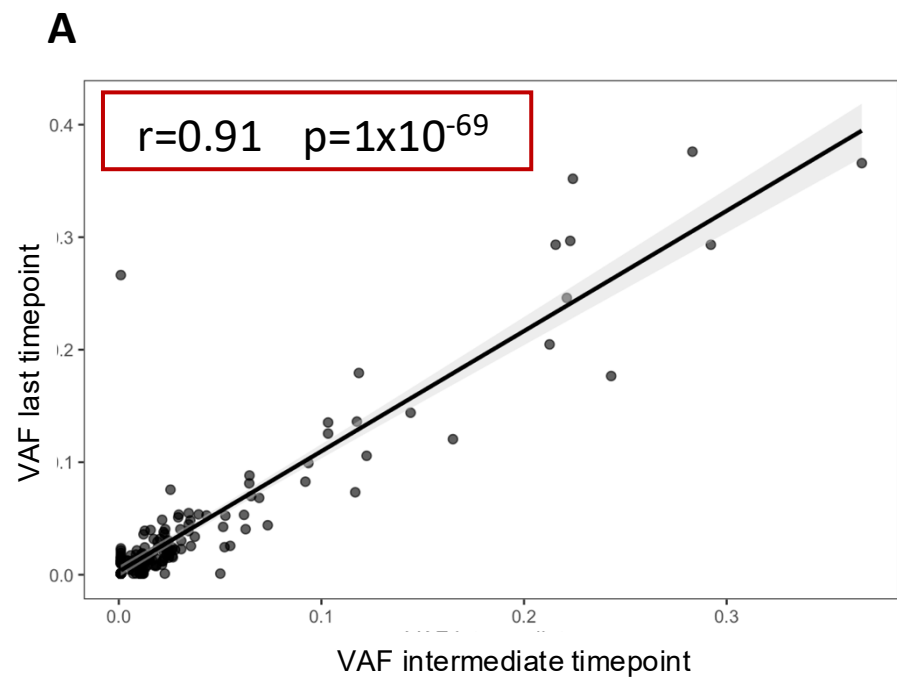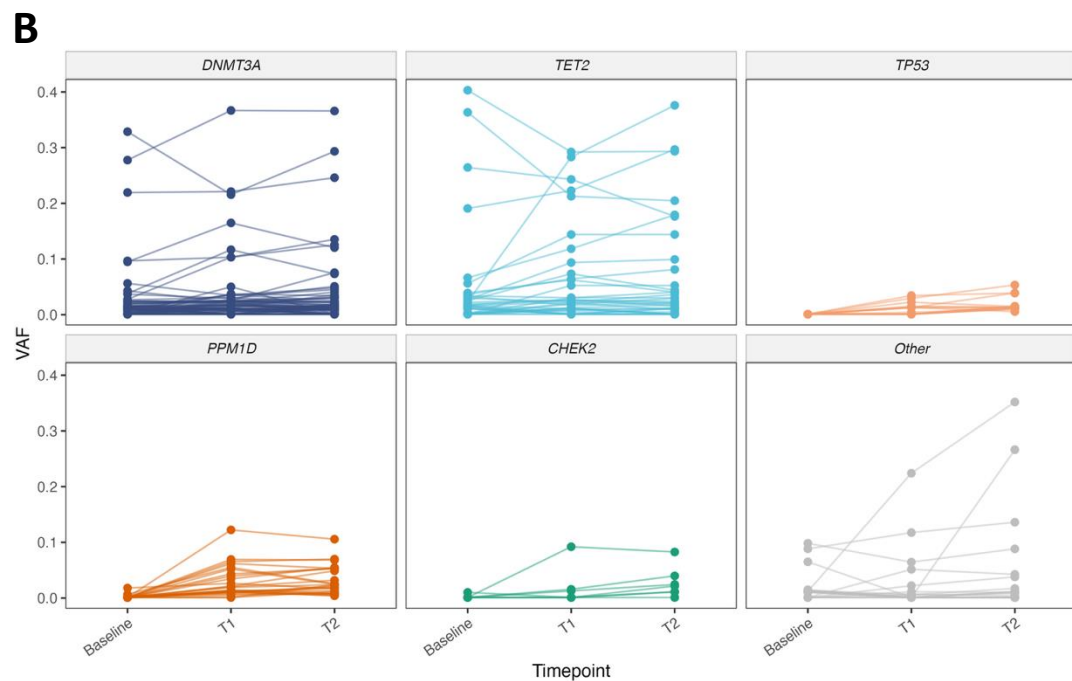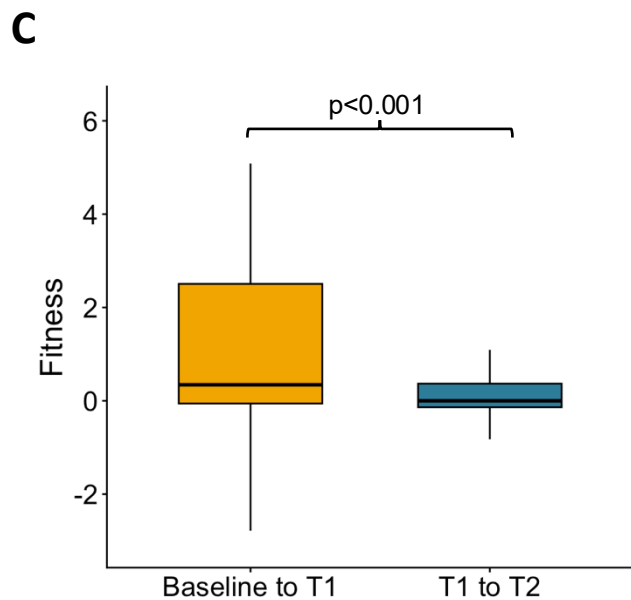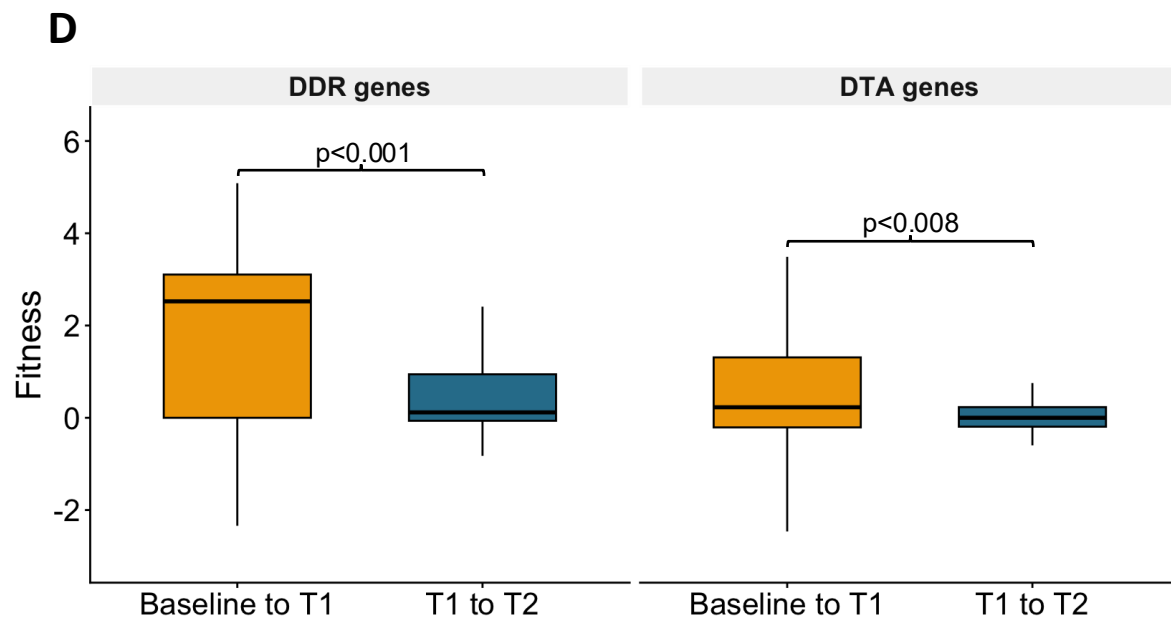

Figure S4

Supplement: Supplementary file 4 — Supporting Information. [file HEM3-10-e70393-s012.pdf]
