# Supplementary material for: Clonal hematopoiesis dynamics influences long‐term outcomes of follicular lymphoma: Results from FIL FOLL12 trial
Source: Hemasphere. 2026 May 20;10(5):e70393. doi: 10.1002/hem3.70393 (PMC13240525; doi:10.1002/hem3.70393)
Supplement: Supplementary file 2 — Supporting Information. [file HEM3-10-e70393-s001.pdf]

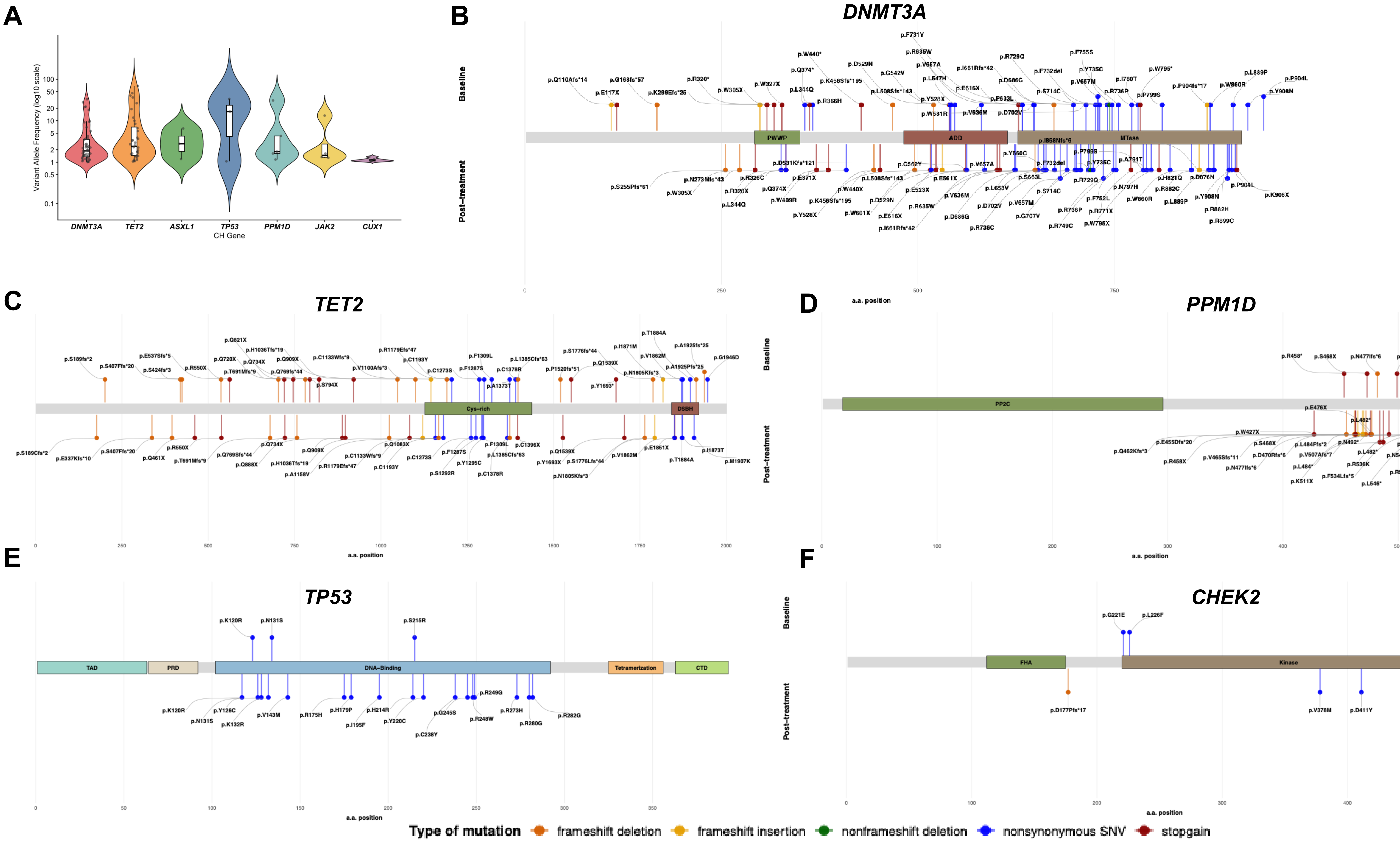
A
Variant Allele Frequency (log10 scale)
100
50
20
10
5
2
1
0.5
0.1
DNMT3A
TET2
ASXL1
TP53
PPM1D
JAK2
CUX1
CH Gene
B
DNMT3A
Baseline
Post−treatment
PWWP
ADD
MTase
0
250
500
750
a.a. position
C
TET2
Baseline
Post−treatment
Cys−rich
DSBH
0
250
500
750
1000
1250
1500
1750
2000
a.a. position
D
PPM1D
Baseline
Post−treatment
p.R458*
p.S468X
p.N477Ifs*6
p.L484*
p.R536K
PP2C
0
100
200
300
400
500
600
a.a. position
E
TP53
Baseline
Post−treatment
p.K120R
p.N131S
p.S215R
TAD
PRD
DNA−Binding
Tetramerization
CTD
p.K120R
p.N131S
p.Y126C
p.K132R
p.V143M
p.R175H
p.H179P
p.I195F
p.H214R
p.Y220C
p.C238Y
p.G245S
p.R248W
p.R249G
p.R273H
p.R280G
p.R282G
0
50
100
150
200
250
300
350
a.a. position
F
CHEK2
Baseline
Post−treatment
p.G221E
p.L226F
FHA
Kinase
p.D177Pfs*17
p.V378M
p.D411Y
p.G466W
p.W495X
0
100
200
300
400
500
a.a. position
Type of mutation
frameshift deletion
frameshift insertion
nonframeshift deletion
nonsynonymous SNV
stopgain

Figure S2
