# Supplementary material for: Clonal hematopoiesis dynamics influences long‐term outcomes of follicular lymphoma: Results from FIL FOLL12 trial
Source: Hemasphere. 2026 May 20;10(5):e70393. doi: 10.1002/hem3.70393 (PMC13240525; doi:10.1002/hem3.70393)
Supplement: Supplementary file 5 — Supporting Information. [file HEM3-10-e70393-s008.pdf]

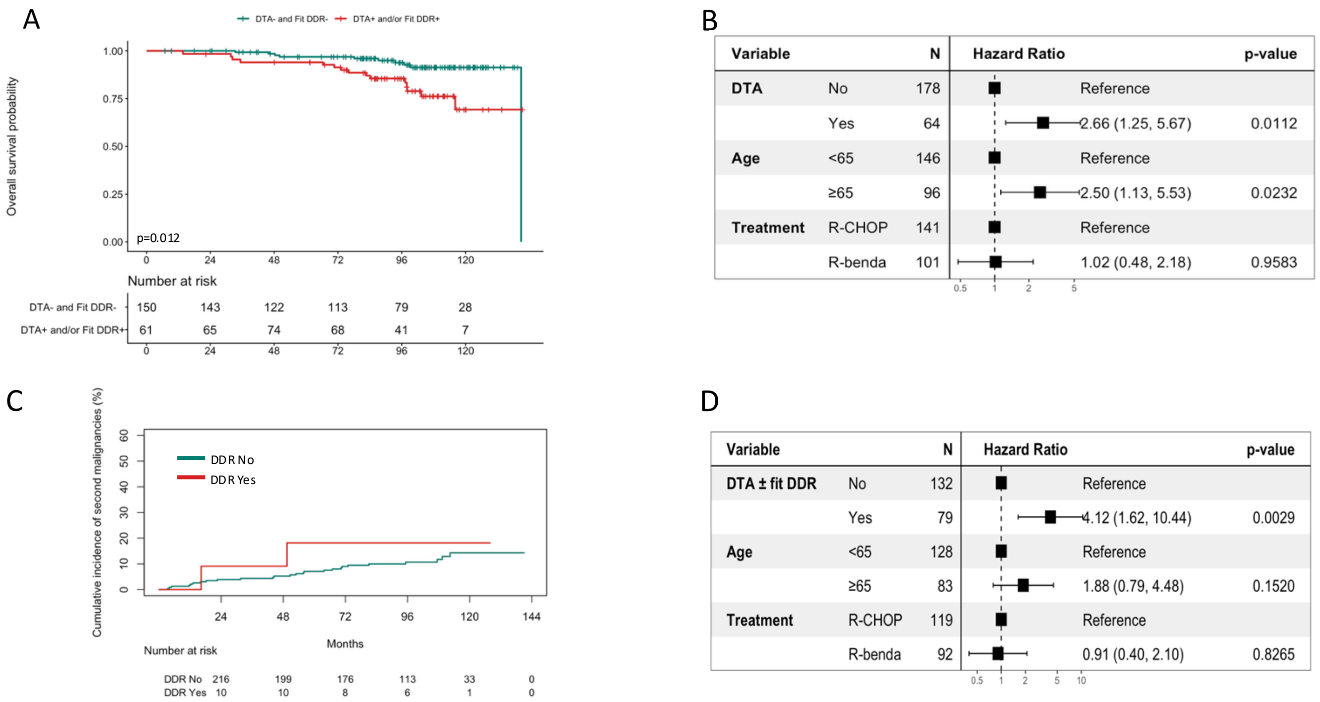
A
DTA- and Fit DDR-
DTA+ and/or Fit DDR+
Overall survival probability
1.00
0.75
0.50
0.25
0.00
p=0.012
0
24
48
72
96
120
Number at risk
DTA- and Fit DDR- 150 143 122 113 79 28
DTA+ and/or Fit DDR+ 61 65 74 68 41 7
B
Variable N Hazard Ratio p-value
DTA No 178 Reference
Yes 64 2.66 (1.25, 5.67) 0.0112
Age <65 146 Reference
≥65 96 2.50 (1.13, 5.53) 0.0232
Treatment R-CHOP 141 Reference
R-benda 101 1.02 (0.48, 2.18) 0.9583
0.5 1 2 5
C
Cumulative incidence of second malignancies (%)
DDR No
DDR Yes
0 10 20 30 40 50 60
24 48 72 96 120 144
Months
Number at risk
DDR No 216 199 176 113 33 0
DDR Yes 10 10 8 6 1 0
D
Variable N Hazard Ratio p-value
DTA ± fit DDR No 132 Reference
Yes 79 4.12 (1.62, 10.44) 0.0029
Age <65 128 Reference
≥65 83 1.88 (0.79, 4.48) 0.1520
Treatment R-CHOP 119 Reference
R-benda 92 0.91 (0.40, 2.10) 0.8265
0.5 1 2 5 10

Figure S5
