# Supplementary material for: Clonal hematopoiesis dynamics influences long‐term outcomes of follicular lymphoma: Results from FIL FOLL12 trial
Source: Hemasphere. 2026 May 20;10(5):e70393. doi: 10.1002/hem3.70393 (PMC13240525; doi:10.1002/hem3.70393)
Supplement: Supplementary file 6 — Supporting Information. [file HEM3-10-e70393-s007.docx]

**SUPPLEMENTARY APPENDIX**

**DNA extraction, quantification and fragmentation**

gDNA was extracted from peripheral blood samples using the Maxwell® RSC Blood DNA Kit and quantified using the Quant-iTTM PicoGreen dsDNA Assay kit (ThermoFisher Scientific, Eugene, OR, USA). PicoGreen is a molecule that binds selectively to double helix DNA and allows to obtain a precise estimate of the amount of DNA. The fluorimetric reading was performed using the Infinite F200 fluorometer (TECAN, Männedorf, Switzerland) using the Magellan software. The fluorimetric readings were obtained at a wavelength of 485 nm in absorption and 530 nm in emission. For quantification, a standard curve was prepared using a DNA of known concentration and performing serial 1:2 scalar dilutions. Quant-iTTM PicoGreen dsDNA Assay kit was used at the 1:200 dilutions. A total of 100 ng of gDNA was used as input for library preparation. The gDNA was fragmented by sonication with the M220 focused ultrasonicator (Covaris® Woburn, MA, USA) before library preparation in order to obtain 300-350 base pair fragments, representing the optimal length for analysis using the MiSeq and NextSeq 550 platforms (Illumina, San Diego, CA, USA). The size of the fragments was checked by using the 2100 Bioanalyzer Instrument with the High Sensitivity DNA kit (Agilent Technologies, St. Clara, CA, USA).

**Library design for hybrid selection**

A custom CAPP-seq gene panel, including coding exons and splice sites of 28 genes (target region: 29710 bp) that are recurrently mutated in CH, has been specifically designed for this project (Table S1).

**Library preparation and sequencing**

Libraries were generated using the KAPA HyperPrep kit (Roche Diagnostics, Pleasanton, CA, USA) and enrichment of regions of interest was achieved using a KAPA HyperChoice probe system (Roche Diagnostics, Pleasanton, CA, USA). Libraries were sequenced using 300-bp paired end runs on MiSeq Illumina platform and 150-bp end runs on NextSeq 550 Illumina platform. The library pool was denatured using 0.2N NaOH. An amount of 6 to 9.5 pM of denatured DNA (for the MiSeq platform) and 1.3 pM (for the NextSeq 550 platform) was loaded into the cartridge.

**Data analysis**

FASTQ sequencing reads were subjected to deduplication by using the FastUniq v1.1. Then, the deduplicated FASTQ sequencing reads were locally aligned to the GRCh37/hg19 version of the human genome assembly using the BWA v.0.6.1 software with the default setting, and sorted, indexed and assembled into a mpileup file using SAMtools v.1. Variant calling of single-nucleotide variants (SNVs) and insertions/deletions (indels) was performed using the somatic function of VarScan2, restricting analysis to nucleotide positions with a Phred base quality score >20. Called variants were annotated using wANNOVAR and subsequently subjected to a multi-step filtering pipeline to retain only high-confidence somatic CH mutations, as detailed below. All retained variants were visually inspected using the Integrative Genomics Viewer (IGV) and interpreted in the context of established CH mutational profiles as reported by Niroula et al.

**Variant filtering**

Called variants were subjected to a multi-step filtering pipeline to retain only high-confidence somatic CH mutations.

- **Functional annotation filter:**
  - Synonymous substitutions and deep intronic variants (>2 bp from exon boundaries) were discarded as non-informative
- **Sequencing quality filter:**
  - Variants with a Phred base quality score <20 were excluded
  - Only variants supported by ≥4 reads on both the forward and reverse strand were retained, ensuring strand-balanced evidence
- **Allele frequency filter:**
  - Variants with a VAF <1% were excluded; a VAF ≥1% was adopted as the minimum threshold for CH mutation calling
- **Variant-level germline filter:**
  - Variants annotated as germline SNPs in the NCBI dbSNP database were excluded; TP53 variants were handled separately and manually curated against the IARC TP53 Database (<http://p53.iarc.fr>)
  - Missense variants lacking somatic annotation in the COSMIC v96 database were discarded
- **Cohort-level noise filter:**
  - A Z-test with Bonferroni correction was applied across the full cohort to flag and remove variants whose frequency across samples was consistent with systematic sequencing artifacts rather than true somatic events

**Variants passing all filters** were classified as putative CH mutations if they belonged to one of the following categories:

- Protein-truncating variants (PTVs), including frameshift indels, nonsense mutations, and canonical splice site alterations
- Missense variants absent from dbSNP and with confirmed somatic annotation in COSMIC v96

**Minimal residual disease analysis**

In order to estimate the potential contamination of gDNA by FL cells, quantification of the IGH::BCL2 rearrangement was also available as previously reported.^1^ For this aim, a sensitivity of 10^-3^ was applied, consistent with the sensitivity of CAPP-seq-based NGS used for CH analysis.

**Clonal fitness and treatment bottleneck effect**

Clonal fitness was inferred from paired VAF measurements in baseline and sequential samples using a logistic growth model derived from the probabilistic framework described by Robertson and colleagues.^2,3^ The model assumes constrained growth within a finite hematopoietic compartment and an approximately constant clone-specific fitness over the observation interval. It enables analytical estimation of growth rates from two time points while incorporating binomial sampling variance related to sequencing depth to quantify uncertainty in VAF measurements.

$$v(t)=\frac{1}{2}\text{ }\frac{1}{1+Ae^{-st}}$$

where $v(t)$ is the VAF at time $t$, $A$ enforces the baseline VAF, and $s$ represents the clonal selective advantage. Fitness values were analytically derived from the two observed VAFs, incorporating binomial variance based on sequencing depth. Standard errors for $s$ were obtained using a delta-method approximation, enabling the construction of two-sided 95% confidence intervals. Clones with $s<-0.25$/year or $s>0.25$/year whose confidence intervals excluded zero were classified as decreasing or increasing, respectively, and all others as stable. Variants passing the filter in one time point were rescued in other time points of the same patient for this analysis. When a variant was below the variant calling limit of CAPP-Seq (0.1%) in the other time points, we assigned a VAF of 0.1% in order to under- rather than over-estimate VAF changes.

To estimate the effective allelic population size during treatment (N_eff), we applied a KL divergence-based bottleneck framework originally developed for viral transmission dynamics and subsequently adapted to model CH clonal dynamics.^4^ In this framework, each CH variant was modeled as an independent clone, and temporal changes in variant allele frequency (VAF) were attributed to binomial sampling.^5^ Unlike clonal fitness, which quantifies the growth advantage of individual clones over time, N_eff captures the patient-level strength of the treatment bottleneck acting on the entire clonal population, reflecting the degree of clonal restriction or preservation of clonal diversity following therapy. For a variant with baseline VAF f_1 and post-treatment VAF f_2, allele counts were assumed to follow:


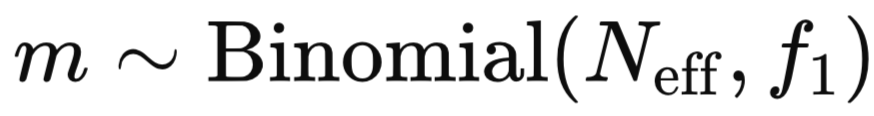


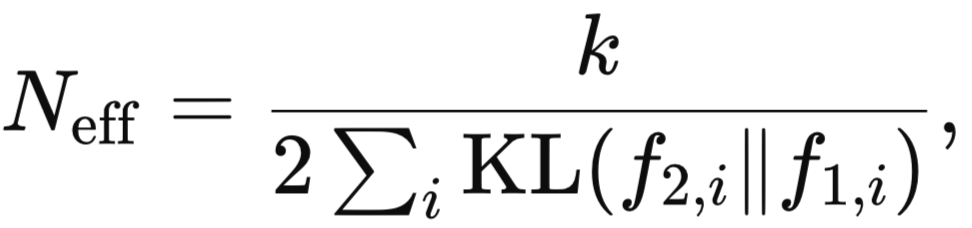
For variants detected longitudinally, $N_{\mathrm{eff}}$ was estimated by maximum likelihood using the Kullback–Leibler divergence between pre- and post-treatment VAF distributions.


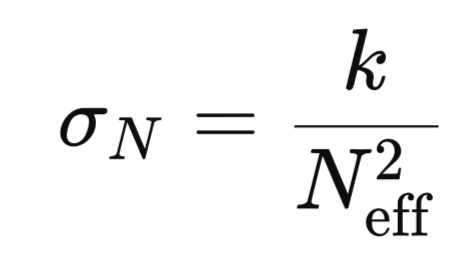
where $k$ denotes the number of shared CH variants. The variance of this estimate was given by

Using the inferred $N_{\mathrm{eff}}$, the likelihood of observing each post-treatment VAF conditional on its baseline value was computed as:


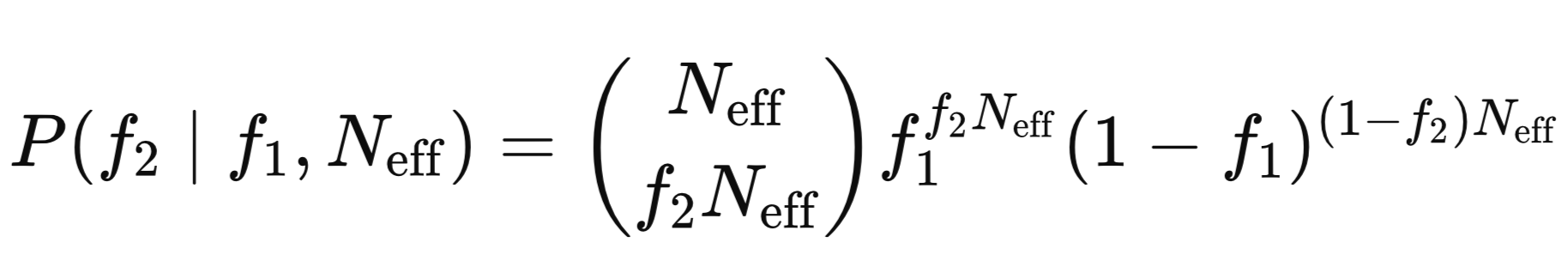


Evolutionary fitness of mutant and wild-type alleles was quantified as the negative base-10 log-likelihood of this probability.

**Statistical analysis**

Survival analysis for progression-free survival (PFS) and for overall survival (OS) was performed by the Kaplan-Meier method and compared between strata using the Log-rank test. The Simon–Makuch method was applied to model fit CH clones as a time-dependent variable. For continuous variables, two group comparisons were performed by Mann-Whitney test, while Chi-square test for categorical variables was used to compare patient characteristics and CH presence. The McNemar test was used to compare CH prevalence at baseline and in paired sequential samples. Associations with the development of second primary malignancies were estimated using both Cox proportional hazards regression, with effect expressed as hazard ratio (HR) with 95% confidence interval (CI) and cumulative incidence function (CIF) analysis by Fine-Gray competing risk modeling with effect expressed as sub distribution HR (sHR) with 95%CI, accounting for death as a competing event. All reported tests were two-sided. Statistics were performed with R version 4.4.2.

**REFERENCES**

1. Ferrero S, Del Giudice I, Galimberti S, et al. Impact of Minimal Residual Disease Analysis in the Era of Rituximab Maintenance in Follicular Lymphoma: Data from “FOLL12” Phase III Trial of the Fondazione Italiana Linfomi. *Blood*. 2024;144(Supplement 1):339-339. doi:10.1182/blood-2024-194832

2. Robertson NA, Latorre-Crespo E, Terradas-Terradas M, et al. Longitudinal dynamics of clonal hematopoiesis identifies gene-specific fitness effects. *Nat Med*. Jul 2022;28(7):1439-1446. doi:10.1038/s41591-022-01883-3

3. Arends CM, Kopp K, Hablesreiter R, et al. Dynamics of clonal hematopoiesis under DNA-damaging treatment in patients with ovarian cancer. *Leukemia*. Jun 2024;38(6):1378-1389. doi:10.1038/s41375-024-02253-3

4. Sobel Leonard A, Weissman DB, Greenbaum B, Ghedin E, Koelle K. Transmission Bottleneck Size Estimation from Pathogen Deep-Sequencing Data, with an Application to Human Influenza A Virus. *J Virol.* 2017;91(14).

5. Arabzadeh M, Tang Y-H, Colin-Leitzinger C, et al. Clonal Hematopoiesis Dynamics and Evolutionary Fitness During Cancer Treatment Impact Clinical Outcomes. *medRxiv*. 2025:2025.08.27.25334581. doi:10.1101/2025.08.27.25334581

**Supplementary figure legends**

**Figure S1: PFS and OS of patients included in the study. (A)** Kaplan-Meir estimates for PFS of the 242 FL patients enrolled in the study. **(B)** Kaplan-Meir estimates for OS of the 242 FL patients enrolled in the study.

**Figure S2. Landscape and distribution of CH-associated mutations at baseline and after treatment.** **(A)** Violin plots showing the variant allele frequency (VAF) distribution of mutations across the most frequently mutated CH genes. **(B–F)** Lollipop plots illustrating the distribution and type of mutations along the protein domains of ***DNMT3A* (B), *TET2* (C), *PPM1D* (D),*TP53* (E),** and ***CHEK2* (F).** Mutations detected at baseline are shown above the protein schematic, while mutations that **emerged post-treatment** are shown below. Each lollipop represents a distinct mutation, positioned according to amino acid location. Colors indicate mutation type: frameshift deletion (orange), frameshift insertion (yellow), nonframeshift deletion (green), nonsynonymous SNV (blue), and stopgain (red). Functional protein domains are annotated along each gene schematic.

**Figure S3. Prognostic impact of CH-associated mutations on progression-free survival (PFS) and overall survival (OS) at trial enrollment.** Kaplan–Meier estimates of PFS and OS are shown according to the presence of CH-associated mutations: DTA **(A, B)**, *DNMT3A* **(C, D)**, *TET2* **(E, F)**, and DDR **(G, H)**. Patients harboring CH mutations are depicted by the red curves, whereas CH-negative patients are shown in blue. Corresponding p-values for between-group comparisons are reported adjacent to each curve.

**Figure S4. Longitudinal analysis of clonal hematopoiesis (CH) dynamics.** **(A)** Pearson correlation between variant allele frequency (VAF) at the intermediate and final time points. Each dot represents an individual mutation; the solid line denotes the linear regression fit. **(B)** Longitudinal VAF trajectories of CH-associated gene mutations (*DNMT3A*, *TET2*, *TP53*, *PPM1D*, *CHEK2*, and others) across baseline, intermediate, and last time points. **(C)** Comparison of clonal fitness across time intervals. Fitness estimates were higher from baseline to T1 compared to the T1 to T2 interval, consistent with early clonal expansion followed by relative stabilization. **(D)** Comparison of clonal fitness across time intervals stratified by gene category (DDR and DTA). In both groups, fitness was significantly higher during the baseline to T1 interval compared to T1 to T2 (DDR: p<0.001; DTA: p=0.008)

**Figure S5. Impact of DTA and DDR mutations on survival and secondary primary malignancies. (A)** Simon–Makuch estimates of overall survival (OS) stratified by the combined presence of DTA and/or fit DDR mutations. DTA^+^ and/or DDR^+^ patients are shown in red, whereas DTA^−^ and DDR^−^ patients are shown in green. **(B)** Multivariable Cox proportional hazards model for the risk of second primary malignancies, including DTA mutation status, age, and treatment regimen (R-CHOP vs R-Benda). Hazard ratios (HRs) with 95% confidence intervals are shown. **(C)** Cumulative incidence of second primary malignancies according to baseline DDR mutation status, with DDR^+^ patients shown in red and DDR^−^ patients in green at trial enrollment. **(D)** Multivariable Cox proportional hazards model for the risk of second primary malignancies, including combined DTA and/or fit DDR mutation status, age, and treatment regimen (R-CHOP vs R-Benda). HRs with 95% confidence intervals are shown.
